# Machine learning enhanced noninvasive transcranial stroke detection using a portable eddy current damping sensor

**DOI:** 10.1016/j.isci.2025.114130

**Published:** 2025-11-19

**Authors:** Haixu Shen, Seyed Mohammadreza Ghodsi, Benjamin Fixman, Bita Ghodsi, Kirsten Azarraga, Narendhar Prasad, Shane Shahrestani, Nerses Sanossian, Gabriel. Zada, Yu-Chong Tai

**Affiliations:** 1 Department of Medical Engineering, California Institute of Technology, Pasadena, CA 91125, USA; 2 Department of Neurosurgery, University of Southern California Keck School of Medicine, Los Angeles, CA 90033, USA; 3 Department of Mathematics, Western University, London, ON N6A 5B7, Canada; 4 Department of Bioengineering, University of Southern California, Los Angeles, CA 90089, USA; 5 Department of Neurosurgery, Cedars Sinai, Los Angeles, CA 90048, USA; 6 Department of Neurology, University of Southern California Keck School of Medicine, Los Angeles, CA 90033, USA

**Keywords:** Bioelectronics

## Abstract

Stroke remains a leading cause of morbidity and mortality globally, with timely diagnosis critical for effective treatment. Current imaging modalities, such as computed tomography (CT) and magnetic resonance imaging (MRI), often face limitations in availability and timeliness, leading to diagnostic delays that worsen patient outcomes. This study presents the development and validation of a portable, noninvasive eddy current damping (ECD) sensor for rapid stroke detection. Building on previous research and optimized through benchtop and phantom studies, this device incorporates advanced coil designs and algorithms to distinguish between hemorrhagic stroke patients and healthy individuals by detecting electrical conductivity variations between normal brain tissue and accumulated blood. Results demonstrate high degrees of accuracy and specificity, promising to enable real-time bedside differentiation of stroke patients. This ECD sensor may expand access to timely stroke care across prehospital, clinical, and remote settings and improve patient outcomes by expediting targeted treatment and rapid triage.

## Highlights

• Eddy current damping sensor enables low-cost and rapid bedside stroke detection
• The sensor features optimized coil and circuit design with KNN classifier
• Benchtop and phantom models validate >90% sensitivity and specificity
• The system is promising for point-of-care integration in diverse medical settings

## Graphical abstract

**Figure:**
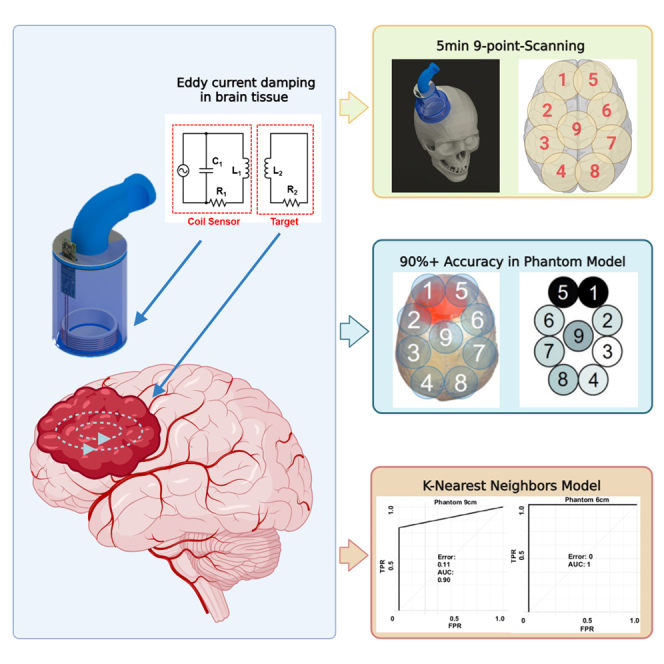

## Introduction

Stroke remains a critical global health issue. Recent data indicate that the lifetime risk of stroke for individuals aged 25 and older has increased from 22.8% in 1990 to 24.9% in 2023, highlighting an 8.9% relative rise even after accounting for competing causes of death.^1^^,^^2^ In the United States, stroke ranks as the fifth leading cause of death and is a major contributor to long-term disability—with about 3% of males and 2% of females reporting significant disability from stroke.^3^ Moreover, survivors often face heightened risks of depression and suicidality, particularly within the first year following the event.^4^^,^^5^

Strokes are broadly classified into two types: ischemic and hemorrhagic. Ischemic strokes result from blood vessel blockages and account for roughly 85% of cases. Hemorrhagic strokes, caused by ruptured blood vessels leading to bleeding in or around the brain, make up about 15% of cases. However, hemorrhagic strokes are associated with higher rates of mortality and long-term disability.^6^ Accurate differentiation between these types is vital since hemorrhagic stroke treatments focus on controlling bleeding and managing intracranial pressure.^7^^,^^8^, while ischemic stroke treatment mainly involves a combination of intravenous thrombolysis and endovascular treatment with mechanical thrombectomy.^9^

Timely diagnosis is essential—often encapsulated in the phrase “time is brain.” Current guidelines recommend brain imaging within 25 min of emergency department arrival; however, only 41.7% of patients met this benchmark in 2017.^10^ This shortfall is due to several factors, including emergency department crowding, transportation delays, and the intrinsic time required for imaging procedures. Although MRI can detect strokes earlier than computed tomography (CT), its limited availability and longer acquisition times restrict its routine use. Furthermore, studies have shown that diagnostic delays are particularly pronounced for ischemic strokes compared to hemorrhagic ones; for example, pediatric cases in England have reported a median delay of 24 h for ischemic stroke imaging versus 3 h for hemorrhagic stroke.^11^^,^^12^

To overcome these challenges, researchers have explored various innovative diagnostic tools, including portable devices based on bioimpedance measurements,^13^^,^^14^ ultrasound^15^^,^^16^^,^^17^ near-infrared spectroscopy (NIRS),^18^^,^^19^^,^^20^ electroencephalogram (EEG),^21^^,^^22^^,^^23^ and microwave imaging.^24^^,^^25^ Despite their potential for rapid, prehospital stroke detection, many of these technologies have not yet been widely adopted into standard clinical practice owing to multiple limitations, including high cost, low accuracy, limited portability. These methods (Table 3) generally provide quick detection and portability with low to moderate cost with decent accuracy yet still facing challenges to be widely applied in clinical settings. Ultrasound-based and NIRS-based techniques usually suffer from shallow penetration due to severe scattering and absorption of ultrasound and IR signals by the skull. Bioimpedance and EEG methods normally require multichannel electrodes and cumbersome wiring, limiting the patient’s mobility and increasing the complexity of scanning. Still, most of these methods are at early stages without wide applications and extensive clinical validations.

In our approach, we introduce an eddy current damping (ECD) sensor designed for rapid stroke detection and classification. This sensor leverages the contrasting electrical conductivity associated with different tissues during stroke onset. For instance, myelin-rich brain tissue exhibits low conductivity (around 0.2 S/m), whereas blood—whether unclotted or clotted—has a higher conductivity (approximately 0.8 S/m).^26^ By identifying regions with abnormal conductivity, the ECD sensor provides critical information that can assist first responders and emergency clinicians in making timely decisions regarding stroke management. This device features major improvements in performance and ease-of use over our previous proof-of-concept device.^8^ In particular, we systematically examined and optimized the circuit, coil and shielding design to achieve a 10-fold increase in signal magnitude and more than twice the penetration depth. The addition of machine learning algorithm enables real-time noninvasive classification^27^ and further elevates diagnostic accuracy to more than 90%. Compared to traditional bioimpedance based technology, our ECD sensor can operate in a completely lead-free manner, eliminating cumbersome wires and electrodes attaching to the skin. The leverage of magnetic field circumvents the interference from skull as magnetic field is minimally absorbed by human tissue. Its portable design makes it particularly valuable in settings such as ambulances and remote locations, potentially expanding access to prompt and specialized stroke care.

Overall, improving the speed and accuracy of stroke diagnosis is paramount for reducing its associated morbidity and mortality. The integration of advanced tools like the ECD sensor into clinical practice holds promise for transforming stroke management by enabling rapid, on-site evaluation and more effective triage to specialized care centers.

## Results

### Sensor principles

**Figure 1:**
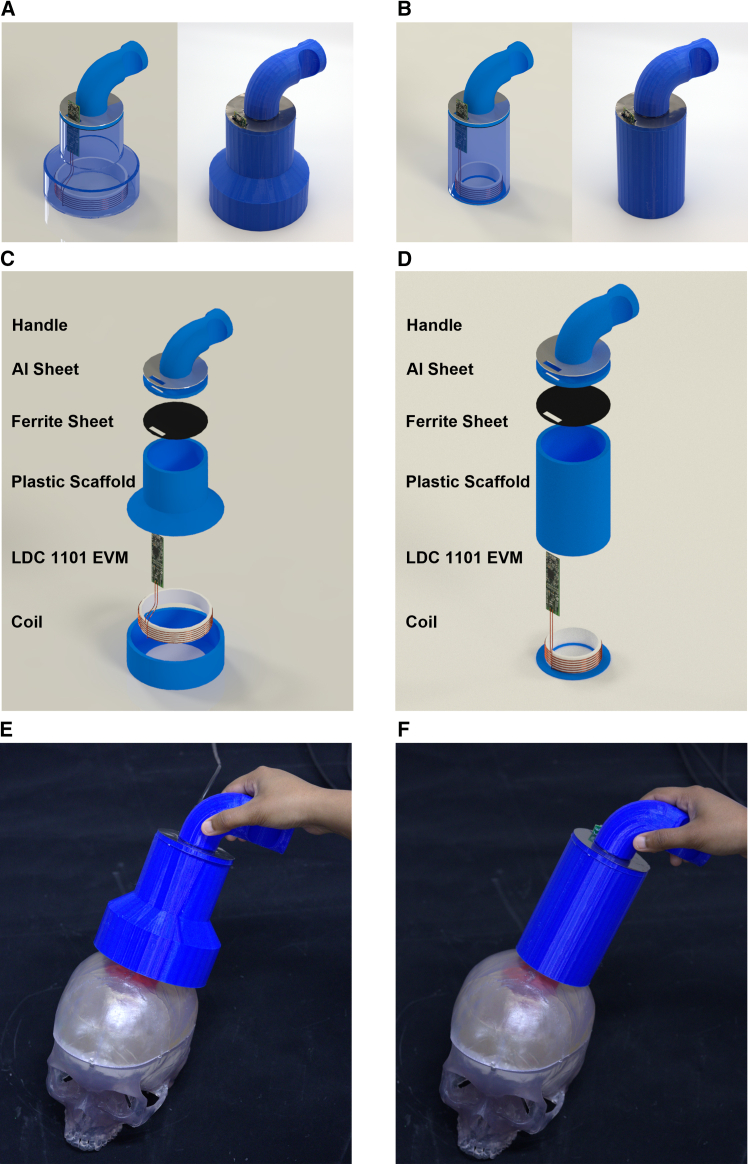
Noninvasive transcranial eddy current sensors for stroke monitoring (A and B) Isometric views of the stroke sensors with 9 cm coil and 6 cm coil. (C and D) Exploded views of the stroke sensors with 9 cm coil and 6 cm coil. (E and F) The stroke sensors scanning a plastic skull model with gelatin simulating normal and hemorrhagic brain tissue.

Figure 1 exhibits an illustrative view of the devices, along with their envisioned clinical use settings. The proposed ECD sensor consists of a 3D printed handle, a plastic scaffold to protect the electronics, grounded thin aluminum sheet (15 μm) for electrostatic shielding, 1 mm ferrite sheet for magnetic shielding, and a Texas Instruments LDC 1101 evaluation board with a customized Litz wire winded coil as the main sensing unit. In this study, two coils with 6 cm and 9 cm diameters both with 6 winds are primarily studied for different detection depths. As shown in Figures 1E and 1F, during detection, the device is intended to scan the surface of the patient’s head. In the presence of a hemorrhagic stroke, higher eddy current is induced within the accumulated blood due to its higher electrical conductivity compared with normal brain tissue, and thus can be detected by the sensor. The resulting color-coded heat maps can be generated and analyzed based on the signal contrast as a preliminary diagnosis of the severity and location of the stroke lesions.

**Figure 2:**
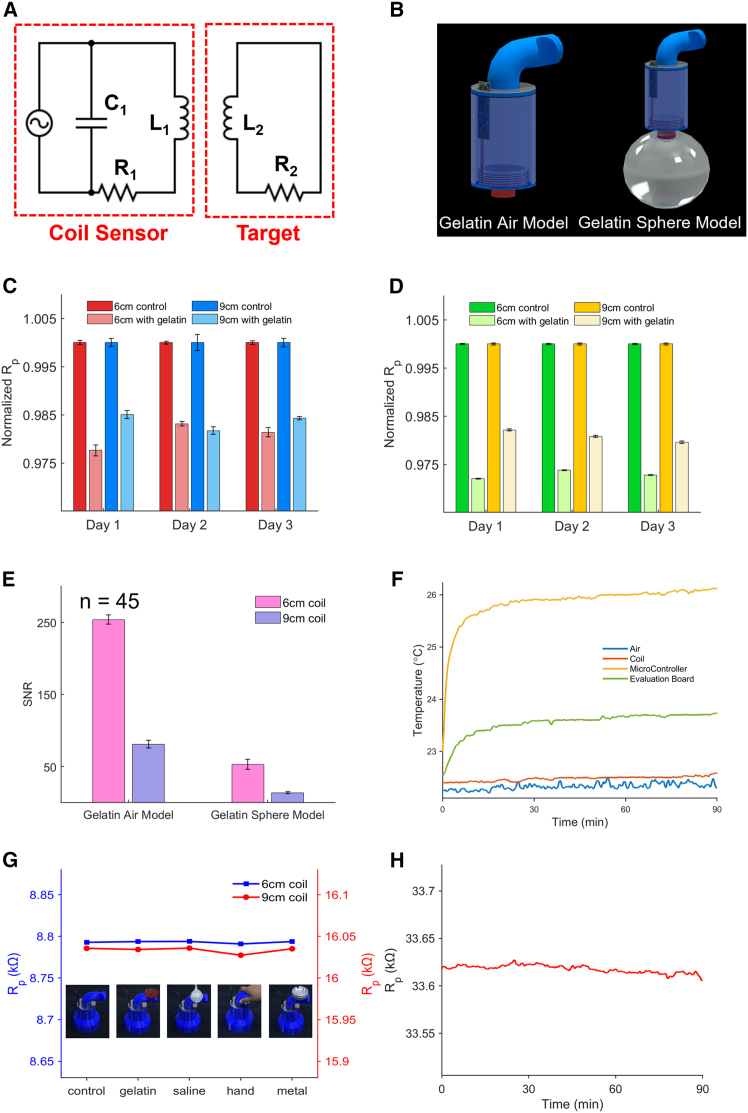
Electrical characterizations of eddy current stroke sensors (A) Equivalent transformer model where alternating current (AC) in the sensor LC (inductor and capacitor) circuit creates a primary magnetic field and induces eddy current on the surface of the conductive subject. The induced eddy current generates a secondary magnetic field counteracting with the primary field and causes change in the inductance and equivalent parallel resistance of the sensor circuit. (B) Animation illustrating the gelatin air model and gelatin sphere model used for signal-to-noise ratio (SNR) characterization. (C) 3-day SNR measurements of sensors using gelatin air model. Data are represented as mean ± SEM. (D) 3-day SNR measurements of sensors using gelatin sphere model. Data are represented as mean ± SEM. (E) Average sensors’ SNR in gelatin air and gelatin sphere models. Data are represented as mean ± SEM. (F) Continuous temperature drift monitoring of the air in the testing room, the sensor coil, the microcontroller, and the evaluation board for 90 min after starting the sensor without conductive objects in the vicinity. (G) The drift in equivalent parallel resistance for both 6 and 9 cm coils by placing gelatin, saline, human hand, and metallic object in the vicinity of the sensor with proper magnetic and electrostatic shielding. (H) The drift in equivalent parallel resistance of the 9 cm coil during measurements mentioned in (F).

As shown in Figure 2A, the ECD sensor can be described using a transformer model, with the conductive target as the secondary coil. When an AC current is applied to the primary coil, according to Ohm’s Law and Faraday’s Law, an eddy current is generated in the conductive target along with an electric field in the reverse direction, counteracting the primary field. The interference from the induced secondary electric field causes a decrease in the inductance of the primary coil, and thus a decrease in the equivalent parallel resistance of the circuit. The equivalent parallel resistance of the circuit can be described by Equation 1, where L_1_, R_1_, C_1_, L_2_ and R_2_ are shown in the circuit diagram, and ω and M are the frequency of the electric field and mutual inductance of the system. From Equation 1, the signal value is positively correlated with the frequency in an ideal setting, but higher frequency increases the resistance of the coil due to skin effect and proximity effect, therefore decreasing the current and the signal. The frequency effect was carefully tested with frequencies ranging from 1 to 5 MHz using a 30 mL saline filled water balloon (see Figure S1 and Videos S1 and S2) to mimic a hemorrhagic lesion and 3 MHz yielded the best experimental results. With comprehensive comparison (see Figures S2 and S3 Table S1), 3.3 μH with 848 pF and 6 μH with 468 pF were chosen for 6 cm coil and 9 cm coil to minimize environmental electromagnetic interferences and parasitic capacitive interferences.

(Equation 1) $$R_{p}=\frac{L_{1}-\frac{\omega^{2}M^{2}}{R_{2}^{2}+{\left(\omega L_{2}\right)}^{2}}L_{2}}{R_{1}+\frac{\omega^{2}M^{2}}{R_{2}^{2}+{\left(\omega L_{2}\right)}^{2}}R_{2}}\cdot \frac{1}{C_{1}}$$

Video S1. Saline SNR measurement using 6 cm coil, related to Figure2

Video S2. Saline SNR measurement using 9 cm coil, related to Figure2

The signal-to-noise ratio (SNR) was tested using 2 models (Figure 2B), the gelatin air model and gelatin sphere model. For the gelatin air model, the sensor was placed still in air without any conductive objects and then followed by directly on top of a 30 mL gelatin filled with saline in air, while R_p_ was continuously recorded for 30 min in each scenario. Signal is defined as the difference in the average R_p_ values while noise is the standard deviation in R_p_ variations. For the gelatin sphere model, the sensor was placed directly over a plastic sphere filled with 0.2 S/m NaCl solution to mimic human brain tissue while data were continuously recorded as baseline. Then, a 30 mL of saline gelatin was attached to the top inner surface of a plastic ball and then the ball was filled with 0.2 S/m NaCl solution to simulate the onset of a stroke. The sensor was placed directly on top of the plastic ball phantom with data recorded. 45 measurements were conducted over a span of 3 days. SNR across 3 days and the average SNR for each scenario are shown in Figures 2C–2E. The 6 cm coil sensor exhibited an SNR of 253.92 ± 6.27 for the gelatin air model and 53.22 ± 6.94 for the gelatin sphere model. The 9 cm coil featured an SNR of 81.25 ± 5.26 for the gelatin air model and an SNR of 13.89 ± 1.38 for the gelatin sphere model. The larger-sized coil is subjected to more environmental noise due to its larger covered area and therefore a lower SNR. The gelatin sphere model involves induced eddy current in the surrounding salt water, therefore elevating the background noise and reducing the SNR. In addition to the intrinsic SNR, the stability of the device subject to heating and human intervention is demonstrated in Figures 2F–2H. To measure the heating effect, three independent thermal couple-based temperature probes were attached to the 9 cm coil, microcontroller, and evaluation board, with an additional one left in the air for background measurement while the device was continuously running for 90 min without any conductive target. Temperature data (Figure 2F) and R_p_ values (Figure 2H) were simultaneously recorded during this period. Despite a slight temperature increase in the microcontroller and the evaluation board, no significant changes in R_p_ values were observed. For human intervention study, objects with varying form factors and conductivities (Figure 2G) were placed on the handle part to test the electrostatic and electromagnetic shielding effect of the sensor. The minor effect observed in Figure 2G is likely due to interference from the subject’s torso. However, the resulting effect is insignificant compared to the signal obtained from the gelatin target. Additionally, this change won’t affect the scan as the operator holds the device in the same manner throughout the scanning. Additionally, COMSOL simulations (Figures S4–S6) confirm negligible interference from human artifacts.

The magnetic field strength of each coil was calculated using Biot-Savart’s law, focusing on the large (9.0 cm diameter) and small (6.0 cm diameter) coils. For the large coil, maximum magnetic flux densities 1.0 cm away (Z = 1.0 cm), 2.0 cm away (Z = 2.0 cm) and 3.0 cm away (Z = 3.0 cm) were 46.74, 38.34, and 28.94 nT, respectively. Similarly, the small coil generated maximum flux densities of 64.34, 43.41, and 26.64 nT at corresponding distances. These data validate the consistency between experimental and simulated values and highlight the following: (1) the ECD sensor’s magnetic fields pose no safety risk to operators or patients and (2) smaller coils exhibit greater sensitivity near the sensor but have more rapid decay as distance increases, while larger coils maintain a more stable and deeper sensing range.

### Benchtop experiments

**Figure 3:**
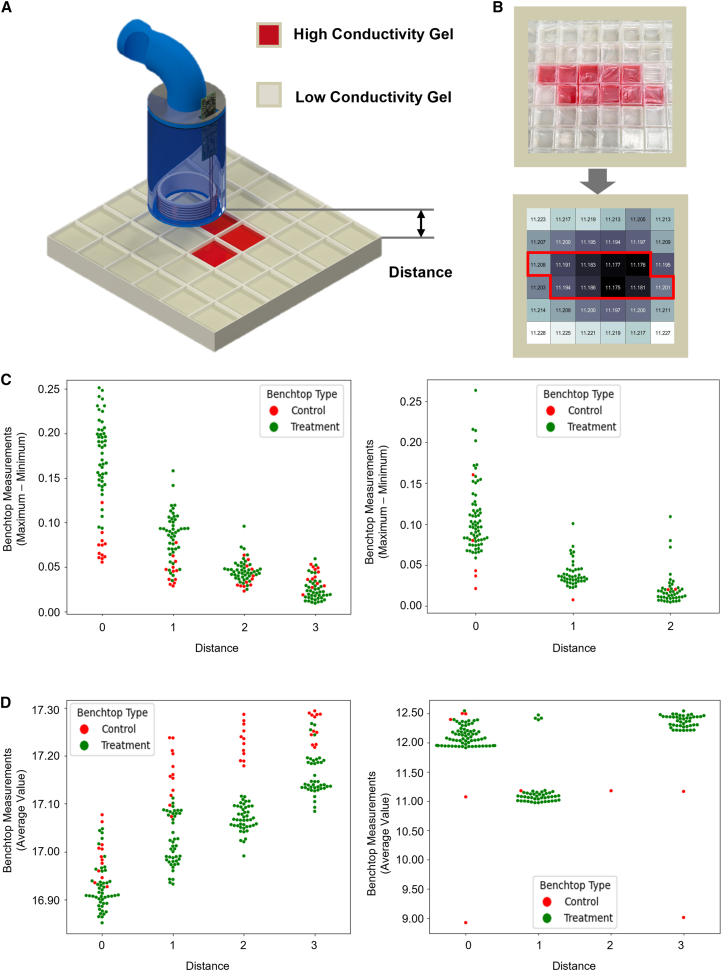
Benchtop recordings of the sensor (A) Illustration of the scanning device positioned over modular cube structure, where red cubes (high conductivity) represent hemorrhage and transparent cubes represent healthy brain tissue. The scanning area is arranged in a 4 × 4 grid with an additional row and column along the edges to reduce edge effects. The device records the signal over each cube for 10 s; the reported value at each coordinate is the average of that 10-s measurement. Scanning all cubes yields a total of 16 data points in a 4 × 4 matrix. (B) Each setup is scanned at distances of 0, 1, 2, and 3 cm (from the bottom of the device to the top of the cube structure) to simulate varying lesion depths in a real-world scenario. (C and D) The distinction between control setups (all transparent cubes) and setups containing at least one lesion (red cube) is shown for different device distances and for two coil sizes (6 and 9 cm). (C) Plots the data range (max – min) per setup, while (D) shows the average signal value across all 16 cubes.

Figure 3 illustrates the benchtop experiments performed using 6 cm and 9 cm diameter handheld sensors over modular cube structures that could be removed or replaced as needed (Figure 3A). Scanning was performed over 4 rows by 4 columns block setups. In order to avoid edge effects, additional rows and columns were placed around the scanning structure (Figure 3B). The block pieces alternated between red-fillings, containing hemorrhagic brain tissue-simulating gels with 0.85 S/m conductivity, and transparent-fillings, containing healthy brain tissue-simulating gels with 0.18 S/m conductivity. Structures with varying configurations, ranging from control (all transparent-filling cubes) to all red-filling cubes, were assembled. In each experiment, the center of the sensor was positioned flat over each cube piece to collect data. For each experiment, the signal was averaged over 10s recordings.

For each setup, 16 data points (4 rows by 4 columns) were recorded. Data from each experiment yielded a 4 by 4 matrix and is demonstrated in a heatmap. Measurements were taken at varying vertical distances (z axis) from the cube structure, specifically at Z = 0.0, 1.0, 2.0, and 3.0 cm. Overall, 52 cube configurations (Figure S7) with repeated control sets (all transparent) were tested for each distance, resulting in a total of 464 4 × 4 matrices (1,856 data points) as the training set for subsequent machine learning algorithm development.

Figures 3C and 3D further demonstrate the distinction between control (green) and treatment (red) setups across varying distances and coil sizes. In Figure 3C, the signal range (maximum - minimum) per setup is mostly wider in treatment cases, particularly at shorter distances, indicating greater signal variability when lesions are present, mostly resulted from the highly diversified test patterns contributing distinctively to the collective ECD. Control setups show narrower ranges, suggesting more uniform signal distribution. Slightly nonuniform distribution of the magnetic field and human artifact during scanning could possibly account for the scattering of the control sets. Figure 3D presents the average signal across all 16 cubes, where treatment setups generally exhibit lower values than controls, especially for the 6 cm coil, highlighting a potentially discriminative feature for lesion detection. The clear separation between red and green points in both panels supports the device’s ability to distinguish setups with and without lesions.

### Phantom experiments

**Figure 4:**
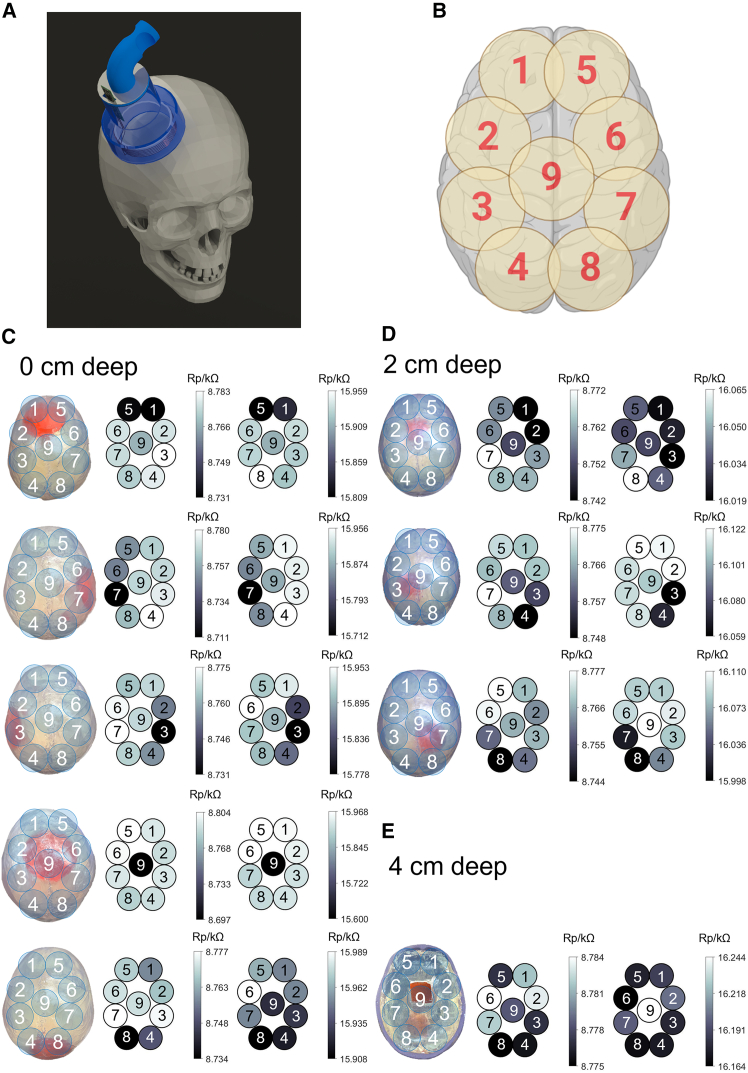
Phantom test results of the eddy current stroke sensor (A) A schematic view of the eddy current sensor scanning a plastic skull with gelatin simulating various stroke scenarios. (B) A top view of the scanning locations covering the entire top half surface of the skull. (C–E) Scanning results for hemorrhagic simulation at different depths: 0 cm (C), 2 cm (D), and 4 cm (E) into the skull. Each circle in the heatmaps directly relates to the scanning point with the same number in the optical images. Color bar represents the equivalent parallel resistance (Rp) of the circuit in kΩ. Lower Rp values indicate higher conductivity, corresponding to hemorrhage-affected tissue. Note that the reconstructed heatmaps are designed to match the orientation of CT/MRI scans and hence are mirror images of the top views and align with the bottom views.

To demonstrate the sensors’ stroke detection capability in 3D models, phantom experiments were conducted as imitations of real stroke patients with lesions in different orientations and depth. During scanning, as shown in Figure 4A, the device was held stationary and loosely touching the top surface of the skull to optimize signal and minimize motion artifact without exerting excessive physical pressure. By Biot-Savart Law, the magnetic field covers the entire area enclosed by the coil, and therefore nine positions (Figure 4B) were carefully chosen for full coverage of the brain. The device was held with more than 70,000 data points recorded for 10 s at each position. Optical photographs of 0 cm lesions and 2 cm lesions (Figures 4C and 4D, the first column) are presented as top views and that of the 4 cm lesion (Figure 4E) is presented as the bottom view owing to their visibility. Heatmaps for 6 cm coil (Figures 4C–4E the second column) and 9 cm coil (the third column) were constructed based on the average Rp value associated with each location, and in an orientation aligned with CT/MRI scans for future clinical studies. Therefore, they are left and right mirror images of the top view optical photographs and the same orientation as the bottom view optical photographs. The points with lower Rp values (darker) indicate higher local conductivity, and hence closer to hemorrhagic lesion. In the optimal instance, the lowest measured conductivity should map exactly the location of the lesion. Nevertheless, factors such as geometric effect, non-uniformity of magnetic field, and loss to the surrounding tissue can significantly affect signal fidelity.

For 0 cm depth (Figure 4C), the lesion is directly attached to the inner side of the plastic skull. Both devices with 6 cm coil and 9 cm coil successfully detected the lesions in 5 settings, with the lowest Rp point directly matching the location of the lesion in the optical image. The 9 cm coil yields larger signal amplitude (∼100 Ω) compared to 6 cm coil (∼50 Ω), while 6 cm coil has slightly better spatial specificity with higher contrast against the surrounding points. Since 9 cm coil has a larger coverage area, it generates more eddy current in the phantom gelatin, causing a higher signal amplitude. On the other hand, higher coverage caused more overlapping between locations, and thus more smeared effect and lower spatial specificity. For 2 cm depth, signal amplitude is reduced for both coils (∼20 Ω for 6 cm coil and ∼50 Ω for 9 cm coil), as eddy current generated on the top gelatin induces magnetic fields counteracting with the magnetic field, thus reducing the magnitude of eddy current generated within the lesions. Decreased specificity and contrast can also be seen and expected, with points surrounding the lesion show a similar Rp value as the lesion point, due to higher eddy current loss in the larger tissue volume. Despite the lower signal magnitude, both sensors were still capable of identifying the locations of the lesions, as points with lower Rp values correspond well with the locations in the top view images. For 4 cm depth, both sensors failed to pinpoint the lesion locations due to the weak effective magnetic field and eddy current generated in the lesion deeply buried by top white gelatin. Therefore, we demonstrated that the eddy current devices are capable of accurate detection of lesions 2 cm deep in the 3D phantom model.

### Predictive performance

**Figure 5:**
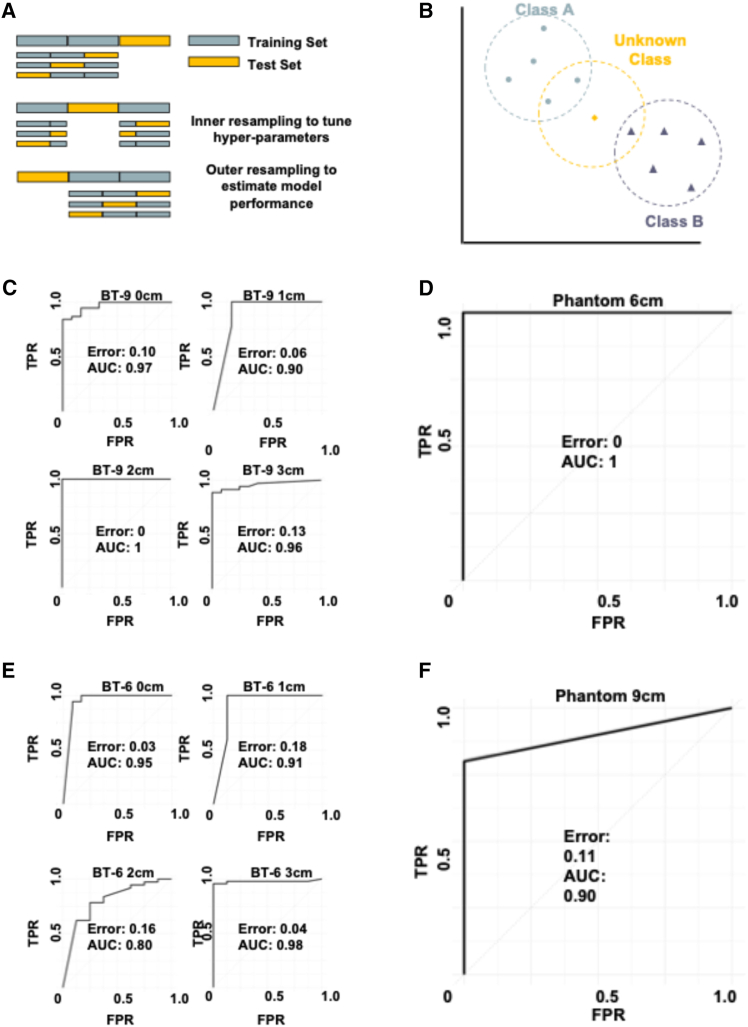
K-nearest neighbor model training and nested cross-validation (A) Schematic overview of K-nearest neighbors model. (B) Schematic overview of nested cross-validation resampling strategy. (C) Nested cross-validation ROC (receiver operating characteristic) curves showing AUC (area under curve) and classification error for the 9 cm benchtop device (BT-9) at each lesion depth (0, 1, 2, and 3 cm). (D) Nested cross-validation ROC curve showing AUC and classification error for the 9 cm device on Phantom across all lesion depths. (E) Nested cross-validation ROC curves showing AUC and classification error for the 6 cm device on benchtop (BT-6) at each lesion depth (0, 1, 2, and 3 cm). (F) Nested cross-validation ROC curve showing AUC and classification error for the 6 cm device on Phantom across all lesion depths.

Following data collection, a K-Nearest-Neighbors Model (KNN) (Figure 5A) was trained to achieve accurate binary classification. We elected to use a KNN model because we anticipated a non-linear decision boundary determined by lesion location. KNN allows each new observation to be classified according to its proximity to lesions in the training data. Provided that the model is trained on lesions in a variety of locations, the unseen data will have “neighbors” to dictate prediction. The dataset with size shown in Table 1, was split (80:20) into training/validation and testing sets. Hyperparameters (K, Distance, Kernal) were systematically optimized on the training/validation data to maximize the model’s ability to discriminate between observations in each class, as measured by receiver operating characteristic-area under the curve (ROC-AUC). Optimal Hyperparameters were subsequently fixed for final evaluation on the holdout test-set.

To rigorously assess model generalizability and minimize bias, nested-cross-validation (Figure 5B) was performed on the training/validation data. Additionally, data collected from the benchtop model were stratified by lesion depth (0 cm, 1 cm, 2 cm, 3 cm) to evaluate device performance separately for both the 9 cm device (Figure 5C) and 6 cm device (Figure 5E). Model performance remained stable across lesion depths for both devices, except for a dip in nested-cross-validation performance for the 6 cm device at 2 cm lesion depth. The ROC curves at each lesion depth display AUC and classification error, providing clear estimates of classification accuracy for both devices at varying lesion depths.

Furthermore, model training and nested-cross-validation were performed on phantom data without depth stratification to approximate real-world clinical performance more closely (Figures 5D and 5F). Finally, the performance metrics of each optimized model evaluated on the independent holdout test-set are summarized in Table 2, highlighting each model’s robust predictive capability, independent of lesion depth.

**Table 1:** Total number of experiments for each device in each setting

Device	Category	Lesion depth	Number of lesions	Number of controls
9 cm device	benchtop	0 cm	48	15
		1 cm	45	15
		2 cm	45	15
		3 cm	44	15
6 cm device	benchtop	0 cm	66	15
		1 cm	46	10
		2 cm	46	10
		3 cm	57	10
9 cm device	phantom	N/A	12	10
6 cm device	phantom	N/A	11	8

**Table 2:** Performance metrics for hold-out test set for both 9 and 6 cm devices tested on benchtop at 0, 1, 2, and 3 cm lesion depth or Phantom

Device	Category	Lesion depth	Accuracy (%)	Sensitivity (%)	Specificity (%)
9 cm device	benchtop	0 cm	85	80	100
		1 cm	100	100	100
		2 cm	100	100	100
		3 cm	92	89	100
6 cm device	benchtop	0 cm	100	100	100
		1 cm	82	77	100
		2 cm	100	100	100
		3 cm	100	100	100
9 cm device	phantom	N/A	100	100	100
6 cm device	phantom	N/A	100	100	100

### Comparison against existing technologies

A summary and comparison of the key performance metrics of standard stroke imaging modalities, emerging technologies and our ECD sensor is exhibited in Table 3 to contextualize the performance of our ECD sensor. Traditionally X-ray based imaging modalities, including non-contrast computed tomography, computed tomography angiography and computed tomography perfusion feature high sensitivity and specificity, moderate scanning time, moderate to high availability and low to moderate cost. However, they’re subject to non-negligible doses of radiation exposure, elevating the risk of cancer. Magnetic resonance-based technologies, such as magnetic resonance imaging with diffusion weighted imaging and magnetic resonance angiography, provide extraordinary diagnostic sensitivity and specificity, yet their limited availability, long scanning time and high cost could impede timely treatment causing brain tissue damage. More portable technologies, namely ultrasound, EEG, bioimpedance and NIRS, facilitate quick detection with low costs and decent sensitivity and specificity, while lack extensive clinical validations and are generally limited to triage patients with severe hemorrhagic stroke due to their limited penetration and signal strength. Our ECD sensor, when validated, offers unique advantages in various aspects including fast measurement and analysis, simple operation, very low cost and wide availability over all the aforementioned modalities. Future optimization and clinical validation could strengthen its specificity, sensitivity and accuracy to a clinically viable level.

**Table 3:** A comparison of core performance metrics of stroke imaging and diagnostic modalities

Modality	Sensitivity/specificity	Scanning time	Availability	Cost	Radiation	Reference
Non-contrast CT (NCCT)	95%–100% within the first 6–24 h	5 min	high	low-moderate	∼2 mSv	Hillal et al.^28^; Heit et al.^29^; Dubosh et al.^30^; Perry et al.^31^
CT angiography (CTA)	>90%	10–15 min	high	moderate	∼3 mSv	Duvekot et al.^32^; Aupongkaroon et al.^33^
CT perfusion (CTP)	sensitivity ∼19%–49% specificity ∼97% to 99%	10–15 min	moderate	moderate	∼2–5 mSv	Benson et al.^34^; Christensen and Lansberg^35^
MRI with diffusion weighted imaging (DWI)	sensitivity ∼88%–97% specificity ∼95%–99%	20–30 min	limited emergently	high	no	Bang and Li^36^; Mullins et al.^37^; Edlow et al.^38^
Magnetic resonance angiography (MRA)	sensitivity ∼95% specificity ∼90%	30 min	limited emergently	high	no	Nederkoorn et al.^39^
Duplex ultrasound (DUS)	sensitivity ∼90%–98% specificity ∼88%–94%	20 min	moderate	low-moderate	no	Nederkoorn et al.^39^; Jahromi et al.^40^;
EEG	sensitivity ∼60% specificity ∼80%	10 min	emerging	low-moderate	no	Wilkinson et al.^23^; Stigt et al.^41^
Bioimpedance	sensitivity ∼93% specificity ∼87% for patients requiring severe stroke triage	5 min	early clinical use	low	no	Young and MacDougall^42^
Near-infrared spectroscopy (NIRS)	sensitivity ∼86% specificity ∼82%	5 min	research and prehospital use	low	no	Zarei et al.^43^
ECD sensor	sensitivity ∼100% specificity ∼100% up to 2 cm deep in phantom model	<5 min	potentially very high	potentially very low	no	this work

## Discussion

In this study, we designed, fabricated, and validated an ECD sensor capable of rapidly detecting hemorrhagic stroke lesions based on variations in electrical conductivity within the brain. The primary motivation for this work was the growing need for portable, real-time stroke diagnostic tools that can function as an adjunct or alternative to standard neuroimaging modalities such as CT and MRI—especially in settings where access to advanced imaging is limited, or timing constraints are critical. The benchtop and phantom experiments we performed demonstrated that the optimized ECD sensor could reliably distinguish between conductive hemorrhagic-simulating gelatin targets and healthy brain tissue–simulating material. While subsequent extensive clinical trials, essential to assess and substantiate its effectiveness in clinical translations, are currently ongoing, our ECD sensor exhibits promising potential to rapidly triage patients to appropriate care settings for further intervention or medical management.

A key aspect of our approach is the reliance on measurable conductivity differences between hemorrhagic lesions and normal brain tissue, an established phenomenon in the bioelectromagnetic field. Hemorrhages exhibit higher conductivity ranges compared to healthy parenchyma, primarily due to the ionic composition of blood. By leveraging this biophysical property, the ECD sensor demonstrates its potential capability of addressing an important gap in stroke management: the need for expedited discrimination of hemorrhagic versus non-hemorrhagic events. While ischemic stroke detection and classification remain beyond the immediate scope of this study, the presented device, when supported by future clinical validations, is poised to integrate seamlessly into the broader clinical workflow, serving as an initial triaging or screening tool for hemorrhagic events.

### Advantages and clinical implications

#### Portability and point-of-care utility

One of the most significant advantages of the ECD sensor lies in its portability. Traditional CT and MRI systems are stationary, expensive, and require specialized operators and infrastructure. They usually cost from hundreds of thousands of dollars to millions of dollars^44^^,^^45^ with power consumptions in the range of kW,^46^ which can be an enormous financial burden to hospitals. In contrast, our proposed handheld ECD sensors can be implemented both at the bedside and in prehospital settings, potentially transforming early stroke detection. With less than 5 min of scanning, the device generates a mapping of the brain tissue conductivity, suggesting the lesion instantaneously. For patients presenting with acute stroke symptoms, immediate hemorrhage differentiation can inform targeted referral to facilities equipped with neurosurgical or endovascular capabilities. With less than 200 dollars cost and mWs power consumption, it can be widely available in almost any scenario. Especially, in resource-limited or rural settings, such a handheld device could substantially reduce transfer times and expedite life-saving interventions.

#### Real-time data acquisition

Our results reveal that the ECD sensor provides near-instantaneous readings, which is of paramount importance in stroke care. Conventional imaging can require significant waiting times due to patient transport, equipment availability, and scheduling constraints.^47^ The rapid detection afforded by the ECD sensor ensures minimal delay in diagnosing hemorrhages, thereby facilitating prompt therapeutic strategies such as blood pressure control, intracranial pressure management, and possible surgical intervention.

#### Versatility of coil designs

We evaluated two different coil sizes (6 cm and 9 cm in diameter), each with distinct advantages. Although the 9 cm coil covered a larger sensing volume and produced higher signal amplitudes, it was more susceptible to background noise and exhibited slightly lower spatial specificity. In contrast, the 6 cm coil produced less absolute signal amplitude but yielded higher spatial resolution. These findings suggest that different coil configurations could be chosen based on specific clinical contexts. For example, the smaller coil may be preferable for localized, high-resolution scans in a hospital setting, whereas the larger coil could be advantageous in a prehospital or field environment where quick, general screening of potential hemorrhages is needed.

### Interpretation of benchtop and phantom findings

#### Depth of lesion detection

Our phantom experiments revealed that hemorrhagic lesions situated up to approximately 2 cm below the phantom’s “skull” surface were detectable, with significant signal differences compared to controls. This limitation in depth penetration is not unexpected, given the fact that tissue absorption and induced eddy currents at shallower levels weaken the energy penetration into deeper regions. Beyond about 2 cm, the signal deteriorated, underscoring the inherent trade-off between detection depth and coil size. In a clinical setting, adult skull thickness, scalp tissue density, and anatomical variations can further influence sensor performance. Additional coil size, inductance and frequency optimizations and calibration for human tissue properties may extend the device’s effective depth range.

In adults, the “overburden” between skin and cortex typically totals about 1.5 cm, with variability by age, sex, and scalp location. Consequently, a 20 mm penetration limit generally affords ∼5–10 mm sampling of parenchyma beneath the cortical ribbon.^48^ From a clinical perspective, this depth budget supports the detection of extra-axial bleeds (epidural, subdural, subarachnoid) and superficial lobar intraparenchymal hemorrhages abutting the cortex, but it is unlikely to capture deep hypertensive hemorrhages in the basal ganglia or thalamus, which are centrally located and several centimeters from the cortical surface. The current device is mainly aimed at cortical/subcortical (lobar) and extra-axial hemorrhages rather than deep ganglionic or thalamic bleeds.

#### Signal-to-noise ratio and environmental factors

Our data demonstrates that environmental noise and coil shielding are critical to achieving a high SNR. Even with substantial electrostatic and magnetic shielding, larger coils inherently capture more environmental electromagnetic interference due to a bigger coverage area. We accounted for temperature-related drifts and anthropomorphic interference—factors likely to be encountered in real-world clinical usage. Despite these confounding variables, the SNR remained sufficiently high to reliably distinguish hemorrhagic-simulating tissue in both benchtop and phantom settings. Further improvements, including incorporation of a faraday cage, automated coil manufacturing for magnetic field uniformity, sophisticated power management and onboard filtering algorithms may further enhance stability and reduce operator-related artifacts.

#### Predictive performance of the classification model

By implementing a KNN model and performing nested cross-validation, we quantified the sensor’s diagnostic accuracy. Crucially, classification performance did not diminish with increasing distance (up to 3 cm in benchtop tests), highlighting the consistency of our device’s measurements across a range of plausible scanning heights. The high AUC values and low classification error rates in the holdout test set confirm the robustness of our approach. However, it is important to emphasize that the exact composition of living brain tissue differs from synthetic gelatin phantoms; thus, *in vivo* validation with patient data remains a necessary step for further model refinement.

### Future directions

The encouraging findings from this study pave the way for several avenues of future research and development.

1. *In vivo* validation: Conduct pilot clinical studies in controlled hospital environments to assess diagnostic accuracy in actual patients presenting with suspected hemorrhagic stroke. Such trials could involve comparing ECD sensor readings to gold-standard imaging results (CT or MRI), analyzing performance metrics, and refining detection algorithms in real-time.
2. Algorithmic enhancements: Incorporate more advanced machine learning or deep learning models, which could integrate not only the ECD sensor’s conductivity readings but also patient-specific clinical parameters such as age, skull thickness, or comorbidities. This might improve sensitivity and specificity, particularly for smaller or deeper lesions.
3. Ischemic stroke classification: Investigate the feasibility of expanding the ECD sensor’s capabilities to differentiate hemorrhagic from ischemic stroke, possibly by modifying coil designs, frequency parameters, or post-processing algorithms to detect subtler conductivity contrasts.
4. Advanced signal processing: Currently, our measurements rely on holding the sensor still and recording data on 9 locations covering the entire head. This method with average Rp value as the signal source leads to decent SNR in the time domain. In the future, more advanced signal processing techniques, especially frequency domain analysis can be researched and leveraged to further enhance the SNR in more complicated settings. It’s potentially viable to reconstruct the hemorrhagic tissue by relying on the contrast in conductivities of different tissues in the frequency domain. To achieve this, extensive studies on the conductivities of related tissues at different frequencies need to be conducted as a database. Additionally, advanced reconstruction algorithms must be developed and validated.
5. Wearable phase array designs: Explore the potential for a wearable device that’s capable of automatic scanning multiple regions of the skull by phase array focusing without moving parts, largely improving scanning speed and reducing operator dependence. Such a platform could integrate multi-coil arrays and advanced reconstruction algorithms for 3D localization of lesions.
6. Integration with telemedicine: Combine the handheld device with telemedicine workflows, enabling first responders to transmit sensor data to neurologists in real time. This approach could accelerate off-site decision making and triaging, especially in geographically isolated regions.
7. Extended applications in neurosurgery and ICU: Beyond immediate stroke detection, the ECD sensor could potentially monitor changes in intracranial conditions (e.g., evolving hematomas, edema) over time in the ICU or operating room. Incorporating real-time monitoring capabilities may allow clinicians to track lesion progression without repeated imaging sessions.

### Limitations of the study

Despite its promising results, this study has several limitations. First, the current benchtop and phantom models with targets of fixed electrical conductivity do not fully reflect the highly variable conductivities in the living organisms. Factors including temperature, pressure, fluid volume fraction, electrolyte concentration and pathological conditions can largely affect the conductivity of brain tissue. While our device’s signal contrast comes from the relative conductivity difference and in theory the classification result can mostly remain unaffected, such variability can affect the capability to interpret the severity of the stroke, leading to overdiagnosis or underdiagnosis. To fully assess and validate the linearity of the sensor’s response and its stability in diagnosing performance across the appropriate range (e.g., 0.1–1.0 S/m), future studies on targets with various conductivities need to be conducted and the collected dataset must be included in the classification algorithm training and validation. More desirably, advanced benchtop and phantom models incorporating variable target conductivities can be developed for a more realistic simulation of the living brain tissues. These advancements can greatly improve the sensor’s stability across physiological and pathological fluctuations, facilitating more accurate diagnosis.

Second, the phantom-based approach, while valuable for proof-of-concept and preliminary evaluation, cannot perfectly replicate the complexities of a living human skull and brain—where variations in geometry, perfusion, temperature, tissue composition, skull thickness, and intracranial pressure could modulate sensor readings.^49^^,^^50^ Future *in vivo* studies or cadaveric models with realistic anatomical and physiological properties should be pursued to confirm the device’s performance under clinically relevant conditions. Our homogeneous benchtop phantoms lack the layered heterogeneity of the human head (high-conductivity scalp, low-conductivity tri-layer skull, meninges, and highly conductive cerebrospinal fluid [CSF]), which strongly shapes current pathways and measured impedance/field patterns. Empirical and modeling studies show that skull compacta vs. diploë differ by an order of magnitude in conductivity and exhibit anisotropy, while CSF (∼1.8 S/m at 37°C) provides a preferential “shunt” that can divert currents tangentially along sulci. Failing to capture these layers can overestimate intracerebral sensitivity and alter apparent depth reach.^50^^,^^51^^,^^52^ Moreover, the dielectric properties of brain, skull, and CSF are frequency- and temperature-dependent (conductivity rises at body temperature by ∼20–25% for CSF, and soft-tissue conductivity generally increases with temperature), so measurements in room temperature, single-frequency phantoms may not translate directly to *in vivo* conditions.^53^ Dynamic physiology further complicates interpretation: cerebral blood volume and flow are pulsatile and autoregulated; blood conductivity varies with hematocrit and aggregation state; and changes in intracranial pressure (ICP)/CSF compliance can modulate intracranial impedance, all of which can introduce time-varying baselines or mimic pathology in superficial sensors.^54^^,^^55^^,^^56^^,^^57^ In addition, subject-specific geometry (scalp-to-cortex distance, regional CSF thickness) and interface effects (hair and skin impedance) materially affect signal amplitude and spatial specificity, underscoring the need for individualized modeling and robust impedance control during acquisition. Ongoing clinical trials are conducted for comprehensive machine learning training and validations to assess and compensate for the dynamic and heterogeneous environments in real human studies and applications.^58^^,^^59^

Third, the focus on hemorrhagic lesions excludes ischemic stroke detection, which constitutes approximately 85% of all strokes. The device is currently intended as a rapid surgical screening tool for intracerebral hemorrhage (ICH), and to triage rapidly to dedicated neurosurgical sites. While the lower conductivity of ischemic lesions may still produce a measurable contrast from normal tissue^60^ additional modifications to the sensor architecture or algorithmic approaches might be necessary to reliably capture these subtler differences. On-going research focusing on improving the power input of the device without adding substantial complexity to the system along with extensive clinical data collection, training and validation is being conducted to assess the capability of detecting the presence of ischemic stroke or infarct. Alternatively, a combined detection approach—initial hemorrhagic screening followed by advanced imaging for ischemic strokes—could become a viable workflow but would require clinical validation.

Fourth, although the classification models using machine learning achieved strong diagnostic metrics in phantom scenarios, these outcomes might differ in clinical environments. Real-world patient variability, motion artifacts, operator differences, and other confounders (e.g., the presence of hair, surgical implants, or scalp electrodes) could affect the ECD sensor’s accuracy. Rigorous validation of the phantom model data against the physiological conditions^61^ are significant for bridging such gaps and extending to future clinical applications. Additionally, appropriate training for operators and iterative data-driven model enhancements (e.g., deep learning approaches) may help mitigate these factors.

Lastly, regulatory pathways for medical device approval, such as Food and Drug Administration clearance in the United States or CE (Conformité Européenne) marking in Europe, require extensive safety, efficacy, and performance testing. Our simulations and benchtop results suggest minimal risks related to electromagnetic exposure, but comprehensive regulatory submissions will necessitate in-depth electromagnetic safety studies, multi-center trials, and demonstration of substantial clinical benefit over standard-of-care imaging.

## Resource availability

### Lead contact

Request for further information and resources should be directed to and will be fulfilled by the lead contact, Haixu Shen (hshen5@caltech.edu).

### Materials availability

This study did not generate new unique reagents.

### Data and code availability

• All data reported in this paper will be shared by the lead contact upon request.
• All codes used in the data analysis and machine learning are available at Mendeley Data: https://doi.org/10.17632/bd5gvn6hvg.1.
• Any additional information required to reanalyze the data reported in this paper is available from the lead contact upon request.

## Acknowledgments

We thank the 10.13039/100000002National Institute of Health (NIH) and the 10.13039/100000065National Institute of Neurological Disorders and Stroke (NINDS) for funding this project (funding number 5R01NS119596). We thank the Medical Engineering Department of California Institute of Technology, University of Southern California Keck School of Medicine, and Department of Mathematics of Western University. The graphical abstract was created in BioRender (Shen, H. [2026] https://BioRender.com/vll4pts).

## Author contributions

Writing – original draft, H.S., S.M.G., B.F., and B.G.; writing – review and editing, H.S., S.M.G., B.F., B.G., N.S., G.Z., and Y.-C.T.; data curation, H.S., S.M.G., B.F., B.G., K.A., and N.P.; validation, H.S., S.M.G., B.F., B.G., K.A., and N.P.; visualization, H.S., S.M.G., B.F., B.G., K.A., and N.P.; resources, H.S., S.M.G., N.S., G.Z., and Y.-C.T.; conceptualization, N.S., G.Z., and Y.-C.T.; methodology, N.S., G.Z., and Y.-C.T.; supervision, N.S., G.Z., and Y.-C.T.; project administration, N.S., G.Z., and Y.-C.T.; funding acquisition, S.S., N.S., G.Z., and Y.-C.T.

## Declaration of interests

Y.-C.T., G.Z., H.S., and S.M.G. have applied for a provisional patent (docket no.: CIT-9161-P2) related to the work described in this manuscript.

## STAR★Methods

### Key resources table

**Table:** 

REAGENT or RESOURCE	SOURCE	IDENTIFIER
**Chemicals, peptides, and recombinant proteins**		

Gelatine Powder Unflavored	Knox	https://knoxgelatine.com/
Sodium Chloride	Sigma-Aldrich	CAS: 7647-14-5

**Deposited data**		

Benchtop and phantom testing raw data	This paper	Mendeley Data: https://doi.org/10.17632/bd5gvn6hvg.1

**Software and algorithms**		

LDC 1101 EVM PC Software	Texas Instrument	https://www.ti.com/tool/LDC1101EVM#description
MATLAB	MathWorks	https://www.mathworks.com/products/matlab.html; RRID:SCR_001622
Python	Python	https://www.python.org/; RRID:SCR_008394
Biorender	Biorender	https://BioRender.com; RRID:SCR_018361
Machine Learning Algorithm for Benchtop and Phantom Expriments	This paper	Mendeley Data: https://doi.org/10.17632/bd5gvn6hvg.1

**Other**		

LDC 1101 EVM Evaluation Board	Texas Instrument	https://www.ti.com/tool/LDC1101EVM#description

### Experimental model and study participant details

This study does not use experimental models, and no animals, human participants, plants, cell lines, or primary cell cultures were involved.

### Method details

#### Sensor signal recording software

LDC1101 EVM PC Software was used as the graphic user interface (GUI) throughout the whole study for device configuration and to retrieve conversion results.^62^ The sensor device was connected to a PC with the GUI installed through a USB cable for communication and data collection. Once launched, Rp+L mode, which continuously streams the equivalent parallel resistance (Rp) and equivalent inductance (L) of the sensor coil, was employed for measuring the equivalent parallel resistance and inductance of the sensor coil. Rp ranges from 6kΩ to 24 kΩ and from 12 kΩ to 48 kΩ were chosen for 6 cm coil and 9 cm coil respectively to achieve the highest resolution. Rp was selected as the main source of signal due to its high sensitivity to losses particularly in low-conductivity materials^63^improved signal to noise ratio^64^and immunity to capacitive coupling.^65^ Once the hardware was placed on the subject and ready for data collection, the enable data log function was activated for 10s and then deactivated with the recorded data saved as a csv file in the specified destination.

#### Sensor tuning

The sensor coil was placed on a wood table with no conductive materials in the vicinity to minimize environmental interferences, and the reading was recorded as the baseline. Next, a 30 mL saline-filled rubber balloon was put on top of the sensor coil with 3D printed PLA plastic spacer of varying heights. The reading with saline target, subtracting the baseline yields the signal generated by the sensor at certain heights. The experiments were repeated with different combinations of capacitances (40pF-7.7 nF), inductances (3.3–25 μH), resonant frequencies(1-5 MHz). Eventually, assessed by their sensing distance and signal amplitude, 6 cm coil with 3.3 μH, 853 pF and 3 MHz resonant frequency, and 9 cm coil with 6 μH, 469 pF and 3 MHz resonant frequency yielded the optimal performances as stroke sensing devices.

#### Brain tissue simulating gel

To simulate the electrical conductivity of healthy brain tissue (0.2S/m) and blood (0.8S/m), ballistic gelatin models were fabricated using gelatin powder (Knox) combined with two different concentrations of sodium chloride. The gelatin powder was dissolved in distilled water using a 9 to 1 weight ratio, and 2% and 0.5% sodium chloride by weight was added to different batches to achieve ∼0.18 S/m and ∼0.85 S/m respectively. Five drops of red food dye were added to the gelatin with higher conductivity to mimic clotted blood. Both mixtures were heated to 50°C and stirred until fully dissolved, then poured into 3D printed grid molds and allowed to cool and solidify. The resulting gelatin phantoms provided consistent electrical conductivity properties for use in sensor testing and simulation of different brain tissue types. The electrical conductivities of both gelatin batches were measured using a standard four-point probe setup.^66^

#### Benchtop experiments

Gelatin simulating normal brain tissue (white gel) and stroke-affected tissue (red gel) were poured into 3D-printed, open-top PLA cubes measuring 1 inch by 1 inch by 1 inch. These cubes were arranged side by side in a 4 by 4 flat grid, creating various combinations of red and white cubes, ranging from entirely white to entirely red configurations. To minimize signal fluctuations due to geometric edge effects, an additional frame of white gel-filled cubes was placed around the perimeter of the 4 by 4 structure. The sensor’s center was positioned directly over each cube in the 4 by 4 grid, and data was recorded for 10 s with a sampling rate of 7 kHz at each location, with the recorded values averaged to reflect the conductivity measurement for that specific coordinate. Data collection was also performed with the sensor positioned at distances of 1 cm, 2 cm, and 3 cm above the 4 by 4 structure, following the same recording procedure.

#### Phantom experiments

The construction of the phantom was based on the average lesion size of clinical hemorrhagic stroke and the corresponding average electric conductivity of normal and affected tissue. An anatomic plastic skull was used as a mold and filled with white gel to simulate normal brain tissue. After completely solidified, the gel was carefully taken out and horizontally cut into 2 cm height pieces. For various stroke scenarios, a cylindrical piece of the white gel with 30 mL volume was selectively removed and replaced with red gel with the same shape to simulate hemorrhagic regions at different coordinates and depths within the head. The sensor was then positioned over nine designated spots on the surface of the head phantom, and signal measurements were recorded for 10s with 7 kHz sampling frequency for each spot. The recorded values were averaged to reflect the conductivity of the phantom at each respective location, providing insight into the spatial variation of conductivity in relation to simulated stroke conditions. The whole process, which will translate to clinical applications in a similar fashion, takes less than 5 min for an adept operator and generates a heatmap reflecting the local tissue conductivity instantaneously.

### Quantification and statistical analysis

#### Machine learning algorithm

For each observation, the 16 (benchtop) or nine (phantom) values of equivalent parallel resistance were recorded as predictor variables (kOhms). Control samples (no lesion) and lesion samples were labeled with a binary outcome “Lesion”, coded as Yes/No. Benchtop data was stratified by lesion depth before undergoing analysis with mlr3.^67^ The data was first stratified by outcome and then split into training/validation and testing sets (80:20). Then, a K-Nearest-Neighbors model was fit to the training/validation data and hyperparameters were optimized using 3-fold cross-validation. The hyperparameters were tuned over a grid of 5 K values (2–6), 10 Kernel choices (rectangular, triangular, epanechnikov, biweight, triweight, cos, inv, Gaussian, rank, optimal), and 5 Distance values (1–5), encompassing 250 different combinations. The hyperparameter combination that resulted in the highest ROC-AUC (Receiver-Operating-Characteristic Area-Under-Curve) was saved for use on the test set. If multiple combinations resulted in the same AUC, the first encountered combination in the tuning space was selected. Nested cross-validation was performed with 3-folds in both the inner and outer loops, using the “*autotuner()*” and “*resample()*” function to assess model performance in a less biased manner. The model with optimal hyperparameters was then used for prediction on the holdout test set.

The total number of lesions and controls used for each model are shown in Table 1.

All analysis was performed in R (v4.3.1) using RStudio (v2024.09.1 + 394) on macOS 15.3.1. The following R packages were utilized: future (v1.34.0), pROC (v1.18.5), data.table (v1.16.0), ggplot2 (v3.5.1), mlr3viz (v0.8.0), mlr3tuning (v0.20.0), paradox (v0.11.1), mlr3learners (v0.6.0), mlr3 (v0.19.0), xlsx (v0.6.5), openxlsx (v4.2.7).

## References

[1] Feigin V.L., Brainin M., Norrving B., Martins S., Sacco R.L., Hacke W., Fisher M., Pandian J., Lindsay P. (2022). World Stroke Organization (WSO): Global Stroke Fact Sheet 2022. Int. J. Stroke.

[2] Gorelick P.B. (2019). The global burden of stroke: persistent and disabling. Lancet Neurol..

[3] Centers for Disease Control and Prevention CDC (2009). Prevalence and most common causes of disability among adults--United States, 2005. MMWR Morb. Mortal. Wkly. Rep..

[4] Harnod T., Lin C.-L., Kao C.-H. (2018). Risk of Suicide Attempt in Poststroke Patients: A Population-Based Cohort Study. J. Am. Heart Assoc..

[5] Towfighi A., Ovbiagele B., El Husseini N., Hackett M.L., Jorge R.E., Kissela B.M., Mitchell P.H., Skolarus L.E., Whooley M.A., Williams L.S. (2017). Poststroke Depression: A Scientific Statement for Healthcare Professionals From the American Heart Association/American Stroke Association. Stroke.

[6] Mallick A.A., Ganesan V., Kirkham F.J., Fallon P., Hedderly T., McShane T., Parker A.P., Wassmer E., Wraige E., Amin S. (2015). Diagnostic delays in paediatric stroke. J. Neurol. Neurosurg. Psychiatry.

[7] Brott T., Bogousslavsky J. (2000). Treatment of acute ischemic stroke. N. Engl. J. Med..

[8] Shahrestani S., Zada G., Chou T.-C., Toy B., Yao B., Garrett N., Sanossian N., Brunswick A., Shang K.-M., Tai Y.-C. (2021). Noninvasive transcranial classification of stroke using a portable eddy current damping sensor. Sci. Rep..

[9] Mosconi M.G., Paciaroni M. (2022). Treatments in Ischemic Stroke: Current and Future. Eur. Neurol..

[10] Kelly A.G., Hellkamp A.S., Olson D., Smith E.E., Schwamm L.H. (2012). Predictors of rapid brain imaging in acute stroke: analysis of the Get With the Guidelines-Stroke program. Stroke.

[11] Adeoye O., Albright K.C., Carr B.G., Wolff C., Mullen M.T., Abruzzo T., Ringer A., Khatri P., Branas C., Kleindorfer D. (2014). Geographic access to acute stroke care in the United States. Stroke.

[12] Ovbiagele B., Nguyen-Huynh M.N. (2011). Stroke epidemiology: advancing our understanding of disease mechanism and therapy. Neurotherapeutics.

[13] Gabriel C., Peyman A., Grant E.H. (2009). Electrical conductivity of tissue at frequencies below 1 MHz. Phys. Med. Biol..

[14] Kellner C.P., Sauvageau E., Snyder K.V., Fargen K.M., Arthur A.S., Turner R.D., Alexandrov A.V. (2018). The VITAL study and overall pooled analysis with the VIPS non-invasive stroke detection device. J. Neurointerv. Surg..

[15] Antipova D., Eadie L., Makin S., Shannon H., Wilson P., Macaden A. (2020). The use of transcranial ultrasound and clinical assessment to diagnose ischaemic stroke due to large vessel occlusion in remote and rural areas. PLoS One.

[16] Herzberg M., Boy S., Hölscher T., Ertl M., Zimmermann M., Ittner K.-P., Pemmerl J., Pels H., Bogdahn U., Schlachetzki F. (2014). Prehospital stroke diagnostics based on neurological examination and transcranial ultrasound. Crit. Ultrasound J..

[17] Schlachetzki F., Herzberg M., Hölscher T., Ertl M., Zimmermann M., Ittner K.P., Pels H., Bogdahn U., Boy S. (2012). Transcranial ultrasound from diagnosis to early stroke treatment: part 2: prehospital neurosonography in patients with acute stroke: the Regensburg stroke mobile project. Cerebrovasc. Dis. Basel Switz..

[18] Liang C.-Y., Yang Y., Shen C.-S., Wang H.-J., Liu N.-M., Wang Z.-W., Zhu F.-L., Xu R.-X. (2018). Chinese Military Evaluation of a Portable Near-Infrared Detector of Traumatic Intracranial Hematomas. Mil. Med..

[19] Robertson C.S., Zager E.L., Narayan R.K., Handly N., Sharma A., Hanley D.F., Garza H., Maloney-Wilensky E., Plaum J.M., Koenig C.H. (2010). Clinical evaluation of a portable near-infrared device for detection of traumatic intracranial hematomas. J. Neurotrauma.

[20] Xu L., Tao X., Liu W., Li Y., Ma J., Lu T., Han B., Liu B., Zhao Y., Li J., Zhao J. (2017). Portable near-infrared rapid detection of intracranial hemorrhage in Chinese population. J. Clin. Neurosci..

[21] Erani F., Zolotova N., Vanderschelden B., Khoshab N., Sarian H., Nazarzai L., Wu J., Chakravarthy B., Hoonpongsimanont W., Yu W. (2020). Electroencephalography Might Improve Diagnosis of Acute Stroke and Large Vessel Occlusion. Stroke.

[22] Michelson E.A., Hanley D., Chabot R., Prichep L.S. (2015). Identification of acute stroke using quantified brain electrical activity. Acad. Emerg. Med. Off. J. Soc. Acad. Emerg. Med..

[23] Wilkinson C.M., Burrell J.I., Kuziek J.W.P., Thirunavukkarasu S., Buck B.H., Mathewson K.E. (2020). Predicting stroke severity with a 3-min recording from the Muse portable EEG system for rapid diagnosis of stroke. Sci. Rep..

[24] Mobashsher A.T., Bialkowski K.S., Abbosh A.M., Crozier S. (2016). Design and Experimental Evaluation of a Non-Invasive Microwave Head Imaging System for Intracranial Haemorrhage Detection. PLoS One.

[25] Persson M., Fhager A., Trefná H.D., Yu Y., McKelvey T., Pegenius G., Karlsson J.-E., Elam M. (2014). Microwave-based stroke diagnosis making global prehospital thrombolytic treatment possible. IEEE Trans. Biomed. Eng..

[26] Beving H., Eriksson L.E., Davey C.L., Kell D.B. (1994). Dielectric properties of human blood and erythrocytes at radio frequencies (0.2–10 MHz); dependence on cell volume fraction and medium composition. Eur. Biophys. J..

[27] Aljanabi R.A., Al-Qaysi Z.T., Suzani M.S. (2024). Deep Transfer Learning Model for EEG Biometric Decoding. Appl. Data Sci. Anal..

[28] Hillal A., Ullberg T., Ramgren B., Wassélius J. (2022). Computed tomography in acute intracerebral hemorrhage: neuroimaging predictors of hematoma expansion and outcome. Insights Imaging.

[29] Heit J.J., Iv M., Wintermark M. (2017). Imaging of Intracranial Hemorrhage. J. Stroke.

[30] Dubosh N.M., Bellolio M.F., Rabinstein A.A., Edlow J.A. (2016). Sensitivity of Early Brain Computed Tomography to Exclude Aneurysmal Subarachnoid Hemorrhage. Stroke.

[31] Perry J.J., Stiell I.G., Sivilotti M.L.A., Bullard M.J., Emond M., Symington C., Sutherland J., Worster A., Hohl C., Lee J.S. (2011). Sensitivity of computed tomography performed within six hours of onset of headache for diagnosis of subarachnoid haemorrhage: prospective cohort study. BMJ.

[32] Duvekot M.H.C., van Es A.C.G.M., Venema E., Wolff L., Rozeman A.D., Moudrous W., Vermeij F.H., Lingsma H.F., Bakker J., Plaisier A.S. (2021). Accuracy of CTA evaluations in daily clinical practice for large and medium vessel occlusion detection in suspected stroke patients. Eur. Stroke J..

[33] Aupongkaroon P., Makarawate P., Chaosuwannakit N. (2022). Comparison of radiation dose and its correlates between coronary computed tomography angiography and invasive coronary angiography in Northeastern Thailand. Egypt. Heart J.

[34] Benson J.C., Payabvash S., Mortazavi S., Zhang L., Salazar P., Hoffman B., Oswood M., McKinney A.M. (2016). CT Perfusion in Acute Lacunar Stroke: Detection Capabilities Based on Infarct Location. Am. J. Neuroradiol..

[35] Christensen S., Lansberg M.G. (2019). CT perfusion in acute stroke: Practical guidance for implementation in clinical practice. J. Cereb. Blood Flow Metab..

[36] Bang O.Y., Li W. (2019). Applications of diffusion-weighted imaging in diagnosis, evaluation, and treatment of acute ischemic stroke. Precis. Future Med..

[37] Mullins M.E., Schaefer P.W., Sorensen A.G., Halpern E.F., Ay H., He J., Koroshetz W.J., Gonzalez R.G. (2002). CT and Conventional and Diffusion-weighted MR Imaging in Acute Stroke: Study in 691 Patients at Presentation to the Emergency Department. Radiology.

[38] Edlow B.L., Hurwitz S., Edlow J.A. (2017). Diagnosis of DWI-negative acute ischemic stroke. Neurology.

[39] Nederkoorn P.J., van der Graaf Y., Hunink M.G.M. (2003). Duplex Ultrasound and Magnetic Resonance Angiography Compared With Digital Subtraction Angiography in Carotid Artery Stenosis. Stroke.

[40] Jahromi A.S., Cinà C.S., Liu Y., Clase C.M. (2005). Sensitivity and specificity of color duplex ultrasound measurement in the estimation of internal carotid artery stenosis: A systematic review and meta-analysis. J. Vasc. Surg..

[41] van Stigt M.N., Groenendijk E.A., van Meenen L.C.C., van de Munckhof A.A.G.A., Theunissen M., Franschman G., Smeekes M.D., van Grondelle J.A.F., Geuzebroek G., Siegers A. (2023). Prehospital Detection of Large Vessel Occlusion Stroke With EEG. Neurology.

[42] Young C., MacDougall D. (2019).

[43] Zarei H., Zarrin A., Janmohamadi M., Saadatipour N., Yarahmadi M., Moeini M., Shams Ardekani S., Safdarian A., Vazirizadeh-Mahabadi M., Babaei M. (2025). Near Infrared Spectroscopy as a Diagnostic Tool for Screening of Intracranial Hematomas; A Systematic Review and Meta-Analysis. Arch. Acad. Emerg. Med..

[44] Saini S., Sharma R., Levine L.A., Barmson R.T., Jordan P.F., Thrall J.H. (2001). Technical Cost of CT Examinations. Radiology.

[45] Westermann R.W., Schick C., Graves C.M., Duchman K.R., Weinstein S.L. (2017). What Does a Shoulder MRI Cost the Consumer?. Clin. Orthop. Relat. Res..

[46] Heye T., Knoerl R., Wehrle T., Mangold D., Cerminara A., Loser M., Plumeyer M., Degen M., Lüthy R., Brodbeck D., Merkle E. (2020). The Energy Consumption of Radiology: Energy- and Cost-saving Opportunities for CT and MRI Operation. Radiology.

[47] Fang J., Yan W., Jiang G.-X., Li W., Cheng Q. (2011). Time interval between stroke onset and hospital arrival in acute ischemic stroke patients in Shanghai, China. Clin. Neurol. Neurosurg..

[48] Bukhari I., Al Mulhim F., Al Hoqail R. (2004). Hyperlipidemia and lipedematous scalp. Ann. Saudi Med..

[49] Hagemann D., Hewig J., Walter C., Naumann E. (2008). Skull thickness and magnitude of EEG alpha activity. Clin. Neurophysiol..

[50] McCann H., Pisano G., Beltrachini L. (2019). Variation in Reported Human Head Tissue Electrical Conductivity Values. Brain Topogr..

[51] Akhtari M., Bryant H.C., Mamelak A.N., Flynn E.R., Heller L., Shih J.J., Mandelkern M., Matlachov A., Ranken D.M., Best E.D. (2002). Conductivities of three-layer live human skull. Brain Topogr..

[52] Baumann S.B., Wozny D.R., Kelly S.K., Meno F.M. (1997). The electrical conductivity of human cerebrospinal fluid at body temperature. IEEE Trans. Biomed. Eng..

[53] Rossmann C., Haemmerich D. (2014). Review of temperature dependence of thermal properties, dielectric properties, and perfusion of biological tissues at hyperthermic and ablation temperatures. Crit. Rev. Biomed. Eng..

[54] Bronzwaer A.-S.G.T., Stok W.J., Westerhof B.E., van Lieshout J.J. (2014). Arterial pressure variations as parameters of brain perfusion in response to central blood volume depletion and repletion. Front. Physiol..

[55] Jaspard F., Nadi M., Rouane A. (2003). Dielectric properties of blood: an investigation of haematocrit dependence. Physiol. Meas..

[56] Kazimierska A., Kasprowicz M., Czosnyka M., Placek M.M., Baledent O., Smielewski P., Czosnyka Z. (2021). Compliance of the cerebrospinal space: comparison of three methods. Acta Neurochir..

[57] Zhbanov A., Yang S. (2015). Effects of Aggregation on Blood Sedimentation and Conductivity. PLoS One.

[58] Formenti D., Ludwig N., Trecroci A., Gargano M., Michielon G., Caumo A., Alberti G. (2016). Dynamics of thermographic skin temperature response during squat exercise at two different speeds. J. Therm. Biol..

[59] Van Hoornweder S., Geraerts M., Verstraelen S., Nuyts M., Caulfield K.A., Meesen R. (2023). From scalp to cortex, the whole isn’t greater than the sum of its parts: introducing GetTissueThickness (GTT) to assess age and sex differences in tissue thicknesses. bioRxiv.

[60] Shu L., Böhm R., Katscher U., Jensen-Kondering U., Scholkmann F., LaManna J., Wolf U. (2022). Oxygen Transport to Tissue XLIII.

[61] Al-Qaysi Z.T., Salih M.M., Shuwandy M.L., Ahmed M.A., Altarazi Y.S.M. (2023). Multi-Tiered CNN Model for Motor Imagery Analysis: Enhancing UAV Control in Smart City Infrastructure for Industry 5.0. Appl. Data Sci. Anal.

[62] TI.com LDC1101 data sheet, product information and support https://www.ti.com/product/LDC1101.

[63] He J., Kong X., Xu Z. (2023). Improving the SNR of UMR sensor using LC resonator. J. Magn. Reson..

[64] Ma M., Liu S., Zhang R., Zhang Q., Wu Y., Chen B. (2024). Non-Destructive Testing of Carbon Fiber-Reinforced Plastics (CFRPs) Using a Resonant Eddy Current Sensor. Sensors.

[65] Chou T.-C. (2022). Wearable Inductive Damping Sensors for Skin Edema Quantification.

[66] Smits F.M. (1958). Measurement of sheet resistivities with the four-point probe. Bell Syst. Tech. J..

[67] Tang X., Bernd B., Raphael S., Lars K., Michel L., Boca Raton F.L. (2025). The American Statistician.

