# Supplementary material for: Machine learning enhanced noninvasive transcranial stroke detection using a portable eddy current damping sensor
Source: iScience. 2025 Nov 19;28(12):114130. doi: 10.1016/j.isci.2025.114130 (PMC12719177; doi:10.1016/j.isci.2025.114130)
Supplement: Document S1. Figures S1–S7 and Table S1 [file mmc1.pdf]

iScience, Volume *28*

Supplemental information

Machine learning enhanced noninvasive transcranial stroke detection using a portable eddy current damping sensor

Haixu Shen, Seyed Mohammadreza Ghodsi, Benjamin Fixman, Bita Ghodsi, Kirsten Azarraga, Narendhar Prasad, Shane Shahrestani, Nerses Sanossian, Gabriel. Zada, and Yu-Chong Tai

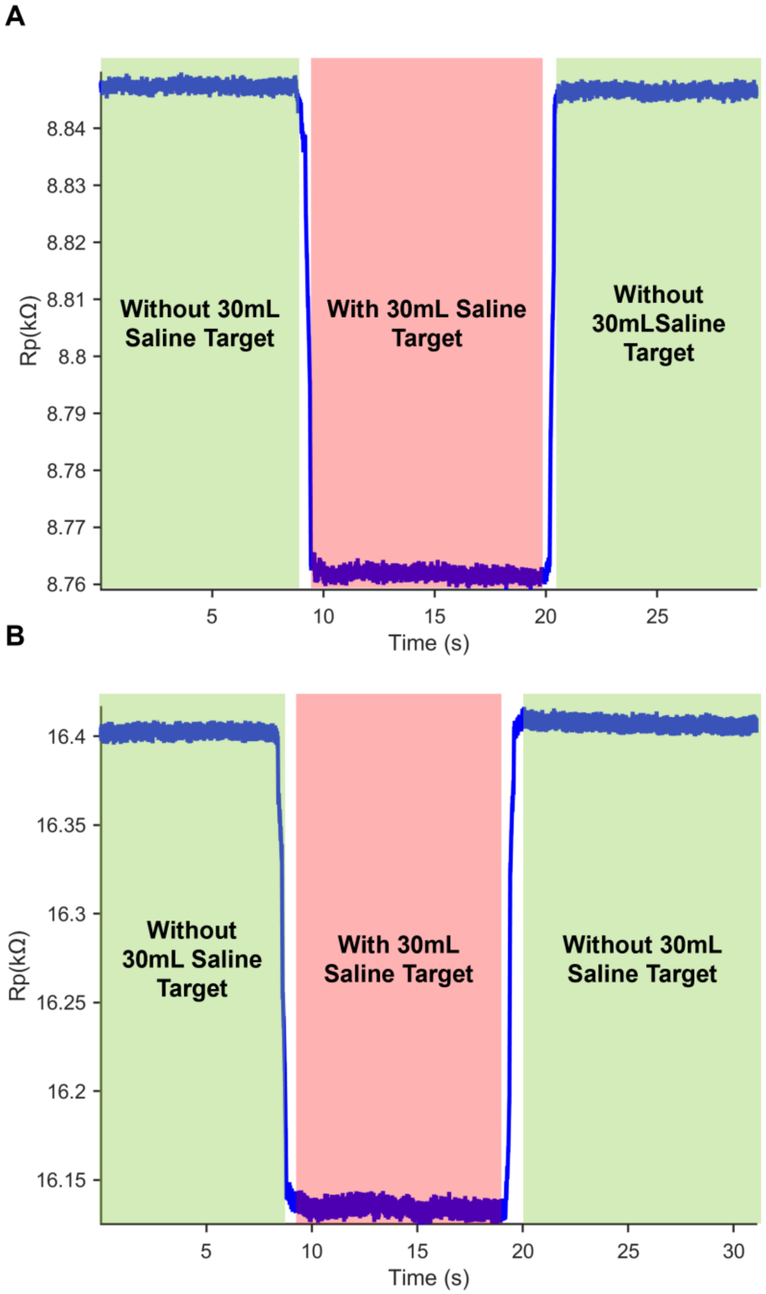

Figure S1. Sample plots of raw signal Rp signal with and without a balloon filled with 30mL 0.8S/m saline solution. Related to Figure 2.
(A) Signal from 6cm coil.
(B) Signal from 9cm coil.

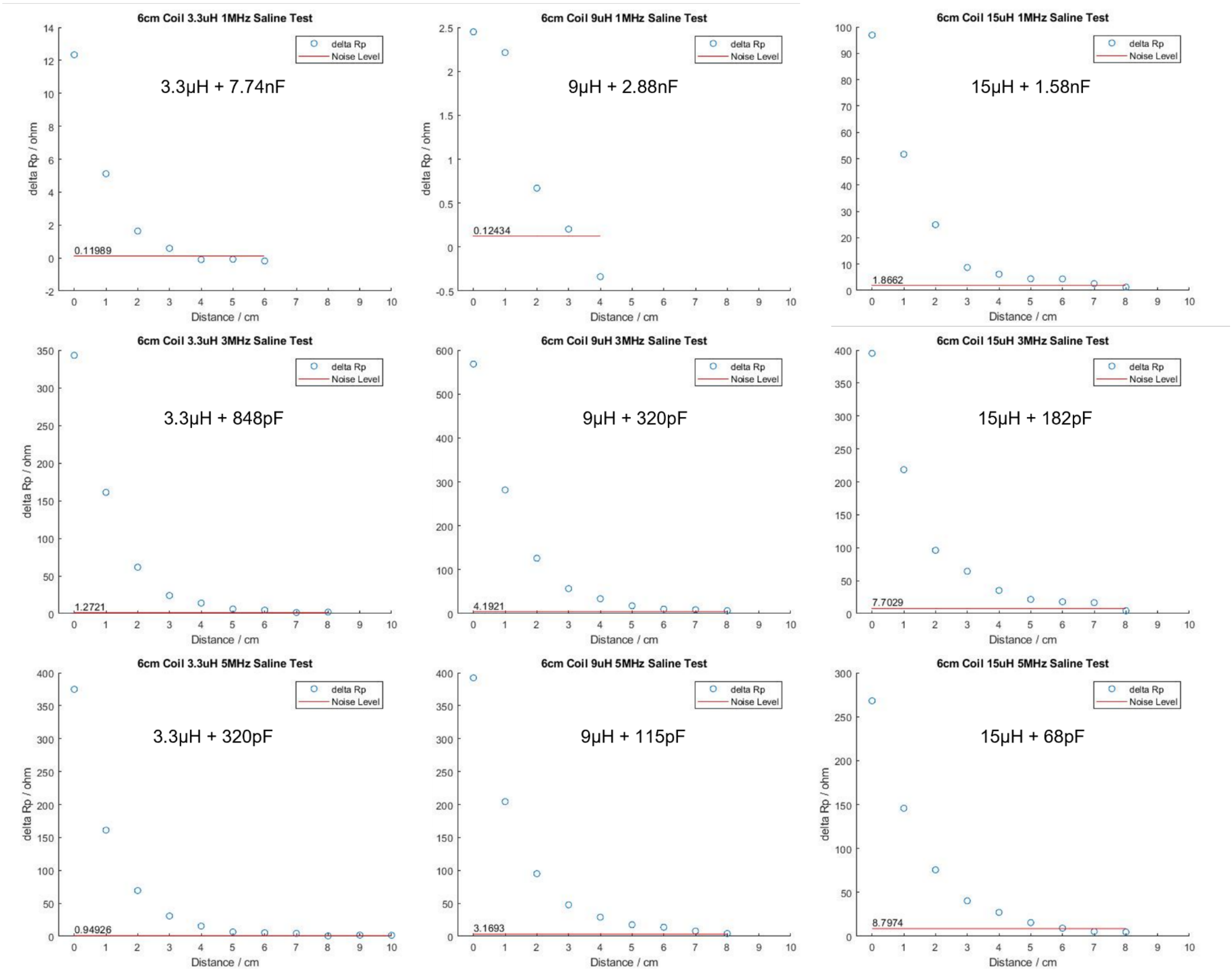

Figure S2. LC tuning curves for 6cm coil. 30mL of saline inside a rubber balloon was used as the target signal source in the atmosphere environment. A SNR of 1 was used as a cutoff for penetration depth. Related to Figure 2.

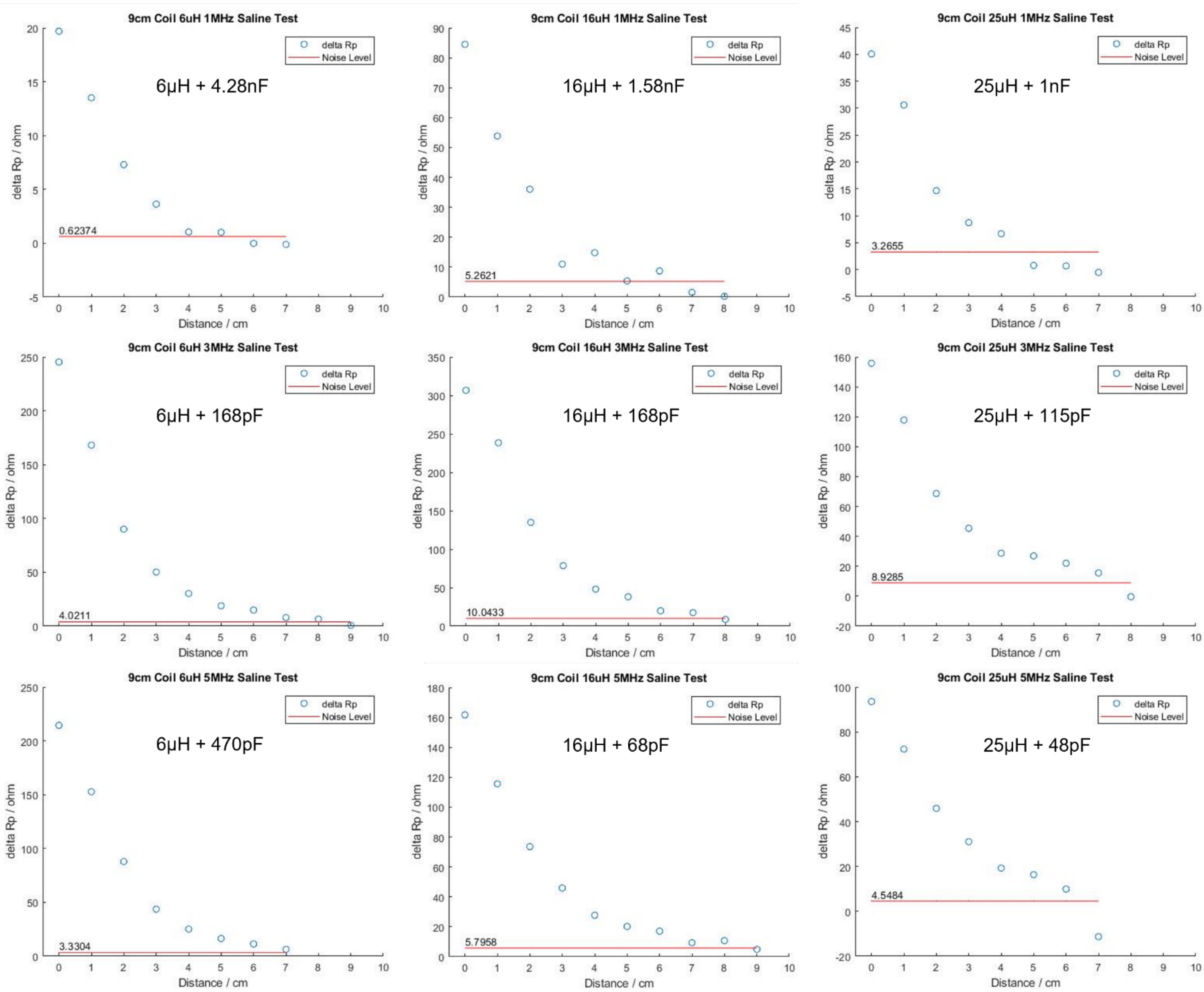

Figure S3. LC tuning curves for 9cm coil. 30mL of saline inside a rubber balloon was used as the target signal source in the atmosphere environment. A SNR of 1 was used as a cutoff for penetration depth. Related to Figure 2.

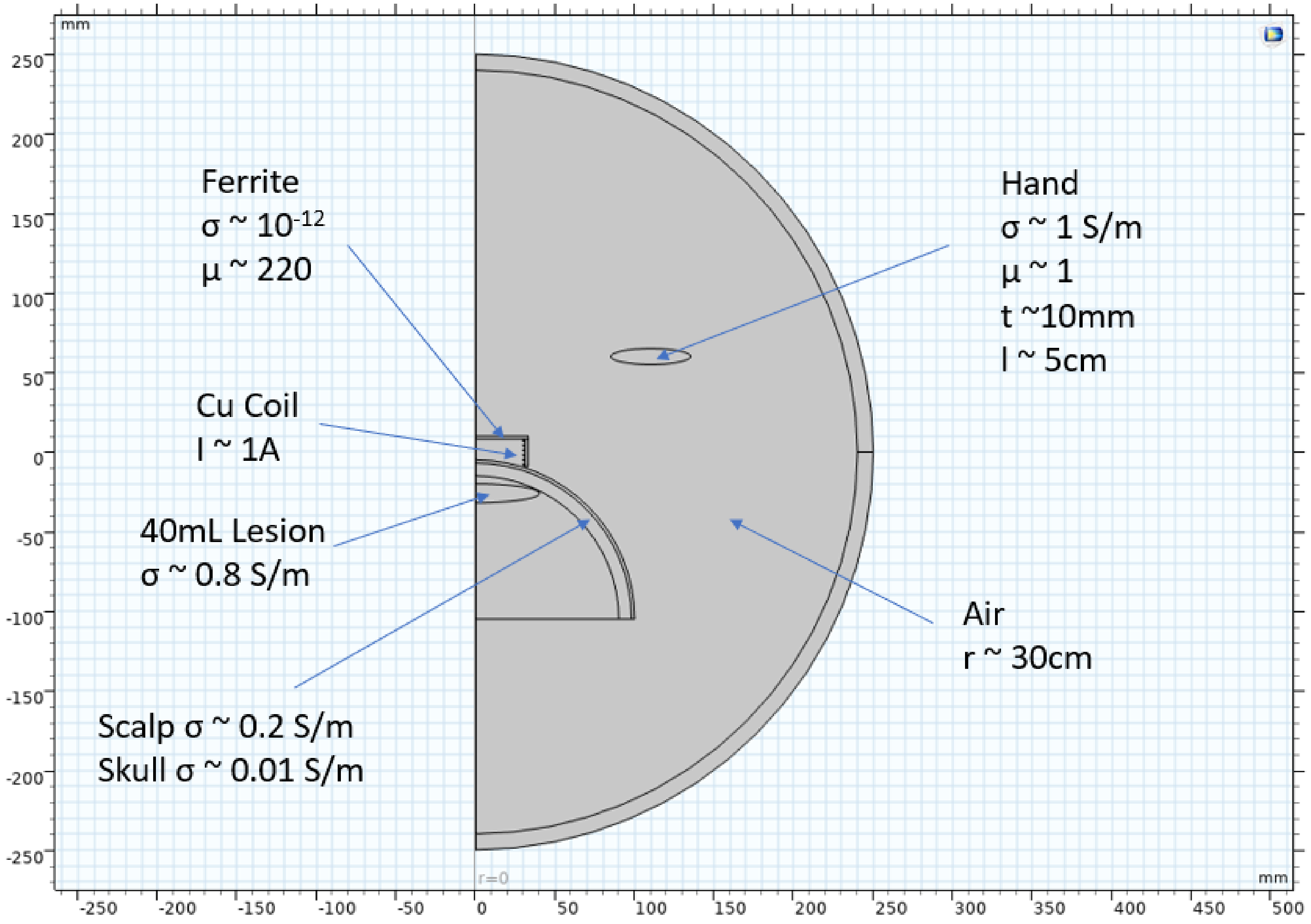

Figure S4. 2D axial symmetric comsol simulation setup for magnetic shielding. Electromagnetic loss to each component was simulated with different thicknesses of ferrite shielding material to study the effect of magnetic shielding. Related to Figure 2.

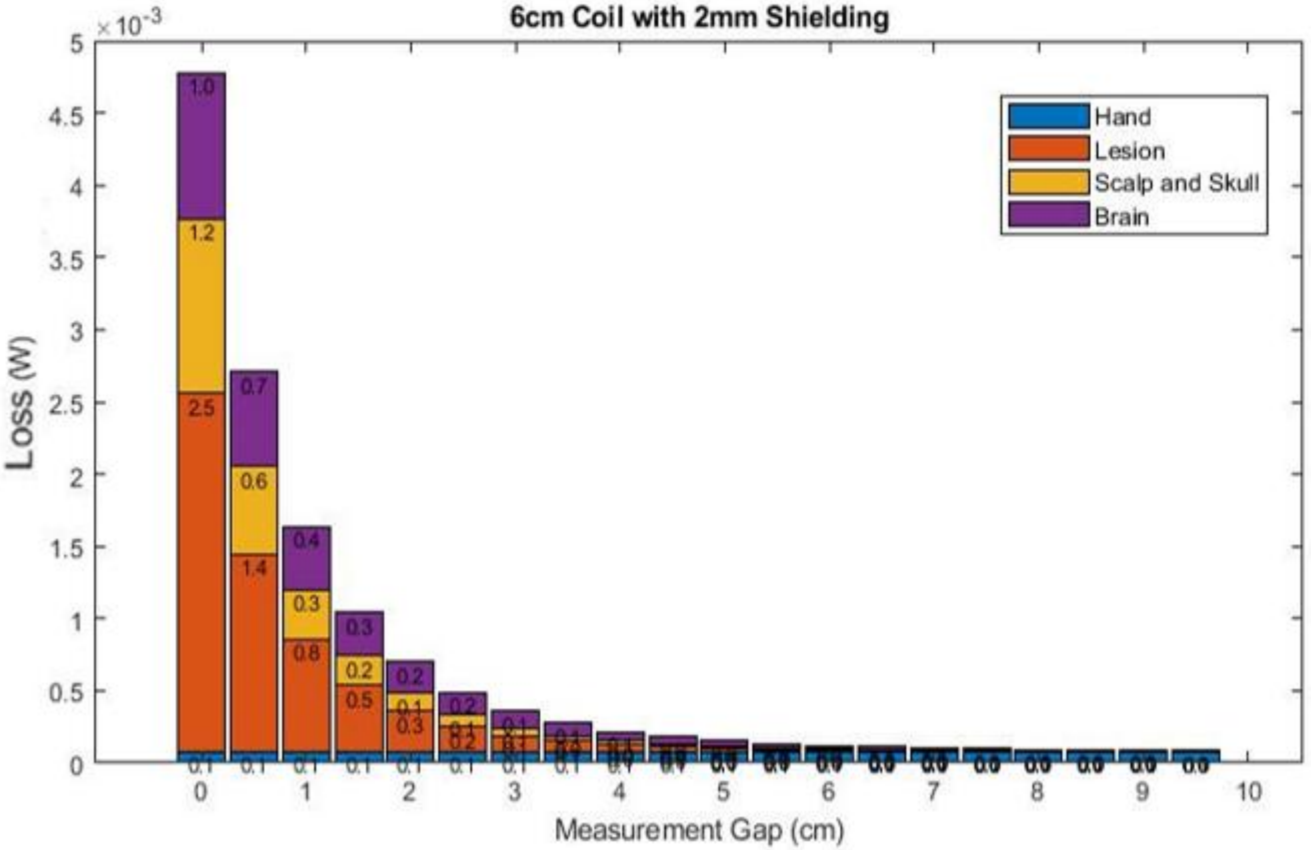

Figure S5. COMSOL simulation of electromagnetic loss for 6cm coil in hand holding the sensor, hemorrhagic lesion, scalp with skull, brain with 2mm thick ferrite shielding material when sensor is placed at 0cm to 9.5 cm away from the phantom model with setup in Figure S4. The magnetic field will induce insignificant amount of energy loss in the hand of the operator when placed directly on top of the phantom head. Related to Figure 2.

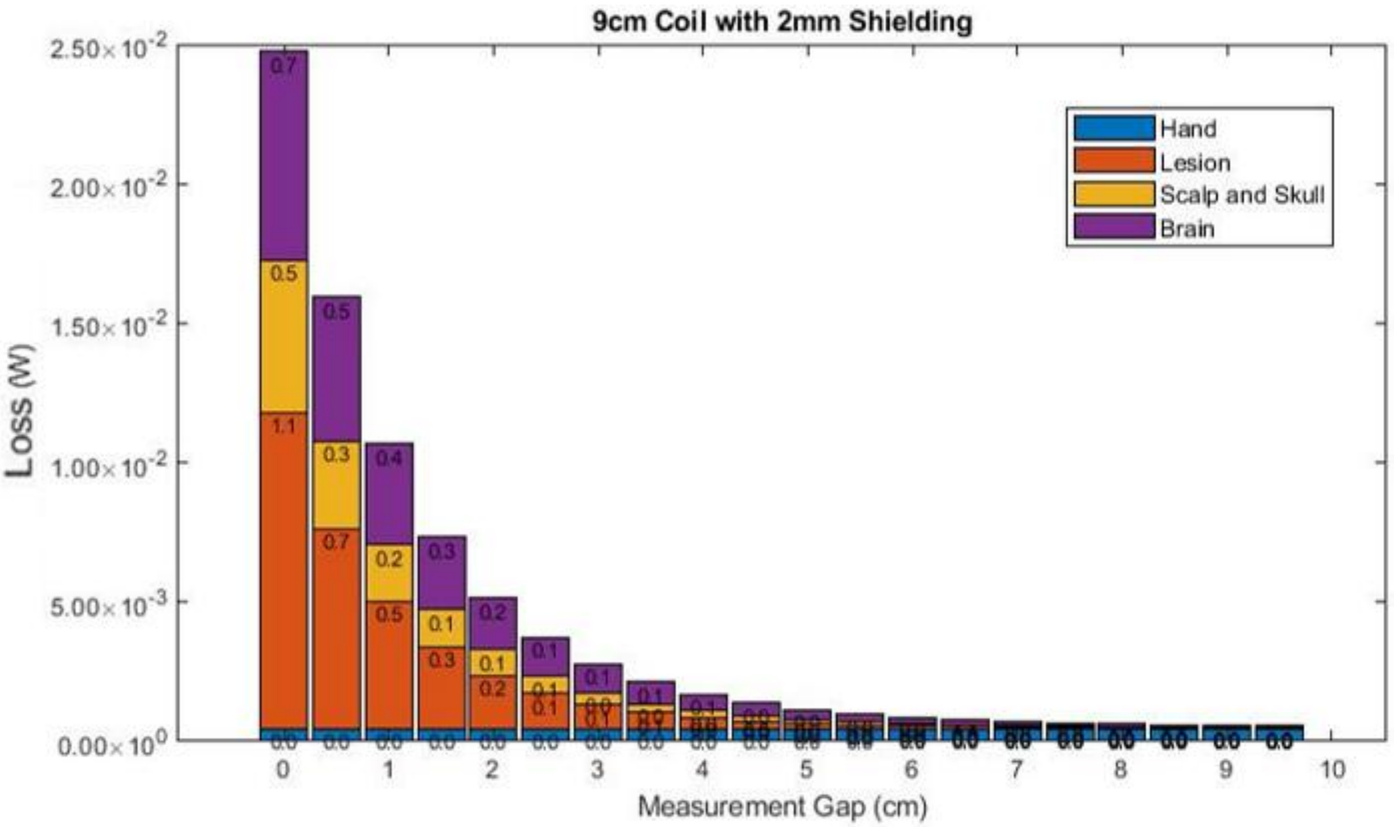

Figure S6. COMSOL simulation of electromagnetic loss for 9cm coil in hand holding the sensor, hemorrhagic lesion, scalp with skull, brain with 2mm thick ferrite shielding material when sensor is placed at 0cm to 9.5 cm away from the phantom model with setup in Figure S4. The magnetic field will induce insignificant amount of energy loss in the hand of the operator when placed directly on top of the phantom head. Related to Figure 2.

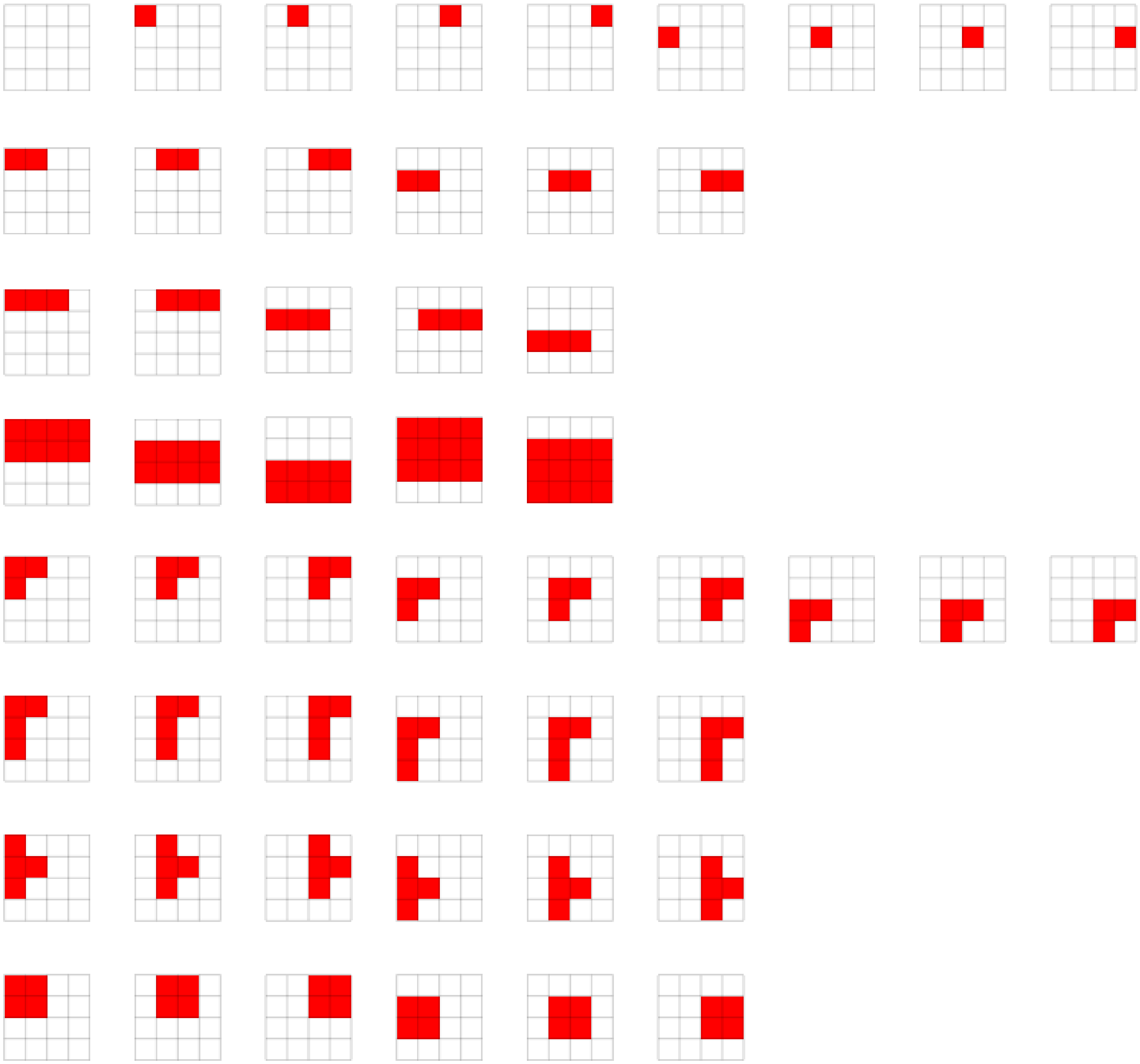

Figure S7. Gelatin configurations scanned in the benchtop study. These configurations were scanned at 0cm, 1cm, 2cm and 3cm distances with 6cm coil and 9cm coil respectively. Related to Figure 3.

Table S1. Summary of maximum signal magnitude for LC combinations Related to Figure 2.

	Inductance	Capacitance	Resonant Frequency	Penetration Depth	Signal magnitude at zero depth
**6cm Coil**	3.3 μH	7.74 nF	1 MHz	3 cm	12 ± 0.1 Ω
	9 μH	2.88 nF	1 MHz	3 cm	2.5 ± 0.1 Ω
	15 μH	1.58 nF	1 MHz	7 cm	95 ± 1.9 Ω
	3.3 μH	848 pF	3 MHz	6 cm	350 ± 1.3 Ω
	9 μH	320 pF	3 MHz	7 cm	560 ± 4.2 Ω
	15 μH	182 pF	3 MHz	7 cm	400 ± 7.7 Ω
	3.3 μH	320 pF	5 MHz	7 cm	370 ± 0.9 Ω
	9 μH	115 pF	5 MHz	7 cm	400 ± 3.2 Ω
	15 μH	68 pF	5 MHz	5 cm	270 ± 8.8 Ω
**9cm Coil**	6 μH	4.28 nF	1 MHz	5 cm	20 ± 0.6 Ω
	16 μH	1.58 nF	1 MHz	4 cm	65 ± 5.3 Ω
	25 μH	1 nF	1 MHz	4 cm	40 ± 3.3 Ω
	6 μH	470 pF	3 MHz	8 cm	250 ± 4.0 Ω
	16 μH	168 pF	3 MHz	7 cm	300 ± 10.0 Ω
	25 μH	115 pF	3 MHz	7 cm	160 ± 8.9 Ω
	6 μH	168 pF	5 MHz	7 cm	220 ± 3.3 Ω
	16 μH	68 pF	5 MHz	8 cm	160 ± 5.8 Ω
	25 μH	48 pF	5 MHz	6 cm	90 ± 4.5 Ω
